# *Plasmodium* cellular effector mechanisms and the hepatic microenvironment

**DOI:** 10.3389/fmicb.2015.00482

**Published:** 2015-05-27

**Authors:** Ute Frevert, Urszula Krzych

**Affiliations:** ^1^Division of Medical Parasitology, Department of Microbiology, New York University School of Medicine, New York, NY, USA; ^2^Division of Malaria Vaccine Development, Department of Cellular Immunology, Walter Reed Army Institute of Research, Silver Spring, MD, USA

**Keywords:** *Plasmodium*, liver, antigen-presenting cells, CD8 T cells, liver lymphatics

## Abstract

*Plasmodium falciparum* malaria remains one of the most serious health problems globally. Immunization with attenuated parasites elicits multiple cellular effector mechanisms capable of eliminating *Plasmodium* liver stages. However, malaria liver stage (LS) immunity is complex and the mechanisms effector T cells use to locate the few infected hepatocytes in the large liver in order to kill the intracellular LS parasites remain a mystery to date. Here, we review our current knowledge on the behavior of CD8 effector T cells in the hepatic microvasculature, in malaria and other hepatic infections. Taking into account the unique immunological and lymphogenic properties of the liver, we discuss whether classical granule-mediated cytotoxicity might eliminate infected hepatocytes via direct cell contact or whether cytokines might operate without cell–cell contact and kill *Plasmodium* LSs at a distance. A thorough understanding of the cellular effector mechanisms that lead to parasite death hence sterile protection is a prerequisite for the development of a successful malaria vaccine to protect the 40% of the world’s population currently at risk of *Plasmodium* infection.

*Plasmodium falciparum* malaria remains one of the most serious health problems globally and long-lasting protective malaria vaccine is desperately needed. The ability to interrupt the clinically silent liver phase of the malaria parasite would prevent an estimated 207 million clinical cases every year, leading to the death of one young African child almost every minute (WHO, 2013). Vaccination with attenuated parasites elicits multiple cellular effector mechanisms that lead to *Plasmodium* liver stage (LS) elimination. While granule-mediated cytotoxicity requires contact between CD8 effector T cells and infected hepatocytes, cytokine mediated parasite killing could occur without cell–cell contact. This review aims to put into context the biology of the pre-erythrocytic stages of *Plasmodium*, the unique immunological and lymphogenic properties of the liver, and recent insight into the dynamic behavior of CD8 effector T cells in the hepatic microvasculature to provide a better understanding of the cellular events involved in the blocking of *Plasmodium* LS development.

## Immunity against Pre-Erythrocytic *Plasmodium* Antigens

While T cell priming against sporozoite antigens is thought to occur in the LNs draining the mosquito bite skin site (Chakravarty et al., 2007), the liver draining LNs are the most likely site of T cell activation against late-LS and early blood stage antigens. However, T cell priming may also occur in the liver itself, for example by direct recognition of infected hepatocytes and or via cross-presentation by the various non-parenchymal antigen-presenting cell (APCs) including hepatic dendritic cell (DCs; Jobe et al., 2009; Crispe, 2011; Bertolino and Bowen, 2015). For an overview on the induction phase of immunity against pre-erythrocytic *Plasmodium* antigens, the reader is referred to recent reviews (Crispe, 2014; Van Braeckel-Budimir and Harty, 2014; Radtke et al., 2015). Here, we focus on the effector phase of the disease and discuss how the various cellular effector mechanisms might operate in the liver, upon first infection of a naïve host leading to disease versus repeated exposure or vaccination resulting in immunity. We present this review in the context of the unique immunological and lymphogenic features of the liver.

## The Liver, a Metabolic Organ with Unique Tolerogenic and Lymphogenic Properties

**FIGURE 1:**
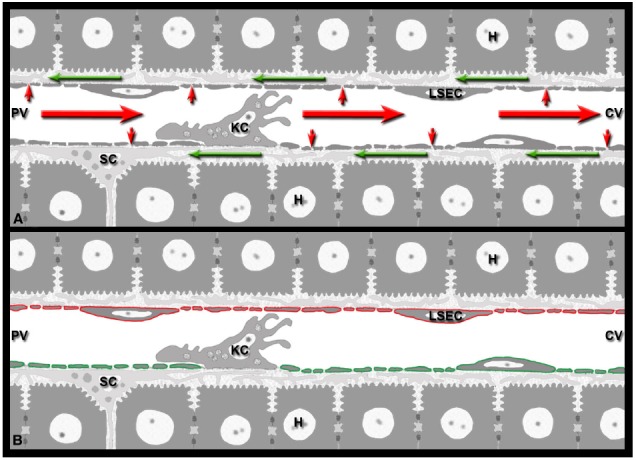
**Immunological implications of the hepatic blood-lymph countercurrent. (A)** The liver generates lymph by filtering blood plasma (small red arrows) through the sieve plates of the sinusoidal endothelial cells (LSEC) into the perisinusoidal space of Disse formed by LSECs and hepatocytes. The lymph (green arrows) flows inside the space of Disse around the perisinusoidal stellate cells (SC) toward the portal field, while the blood (red arrows) continues its path in the opposite direction, from the portal venule (PV) to the central venule (CV). **(B)** LSECs represent the blood-lymph barrier of the liver: they express both the vascular marker PECAM-1 (red) and the lymphatic marker LYVE-1 (green). The two markers are depicted separately for clarity.

The liver is known as a lymphatic organ with unique immunological properties (Knolle and Limmer, 2001; Sheth and Bankey, 2001; Bertolino et al., 2002; Mackay, 2002; Racanelli and Rehermann, 2006; Crispe, 2009). Its tolerogenic properties, necessitated by continuous natural exposure to innocuous food antigens and commensal microbial products from the gastrointestinal tract, are now widely recognized (Racanelli and Rehermann, 2006; Crispe, 2009; Jenne and Kubes, 2013). It seems likely, therefore, that by choosing the liver as the initial site of multiplication, *Plasmodium* is able to exploit the tolerogenic properties of the liver (Frevert et al., 2006; Crispe, 2011; Bertolino and Bowen, 2015). Less appreciated is the generation of lymph in this large metabolic organ. Plasma flows continuously through the sinusoidal sieve plates and enters the space of Disse (Figure 1). Once in the perisinusoidal space, the lymph travels in a retrograde fashion around the sinusoids toward the periportal space of Mall (Reid et al., 1992). Despite more than half of the lymph of the entire body being of hepatic origin (Henriksen et al., 1984; Magari, 1990; Trutmann and Sasse, 1994; Ohtani and Ohtani, 2008), the contribution of lymph formation to liver immunology has been surprisingly underappreciated to date (reviewed in Frevert and Nacer, 2013). By influencing cytokine dissemination, the unique hepatic blood-lymph counterflow principle has important implications for the effector phase of immunity against *Plasmodium* LS.

Most non-parenchymal liver cells can function as APCs (Jobe et al., 2009; Crispe, 2011), inducing either tolerance or enhanced immune responses amongst liver T cells. The state of immune tolerance rather than immune activation is the more typical and frequent condition under the steady state. It prevents liver pathology arising from a constant inflow of bacterial and other microbes from the gastrointestinal tract and blood-borne antigens from the systemic circulation. The state of tolerance observed in the liver is maintained by the production of anti-inflammatory cytokines, in particular IL-10 (Knolle et al., 1995), and other modulators such as PGE2 produced by Kupffer cells (KCs) via ligation of TLR4. Together with nitric oxide, these mediators down-regulate antigen uptake by liver sinusoidal endothelial cells (LSECs) and DCs, which leads to a decreased T cell activation (Roland et al., 1994; Groux et al., 1996). However, KCs have also been shown to play a role in fighting infections by producing IL-12 and IL-18 and upregulating MHC I and II molecules as well as the co-stimulatory molecules CD40 and CD80 needed for activation of T cells to produce IFN-γ (Burgio et al., 1998). It appears therefore that signals received by KCs propel them either into poor and ineffective or efficient APC, promoting a reversal of immune tolerance. Tolerance induction or maintenance is also a particular property of the LSECs (Berg et al., 2006; Ebrahimkhani et al., 2011) with their typical fenestrations. Although LSECs express MHC I and II molecules as well as costimulatory molecules involved in T cell activation, they produce IL-10 upon TLR4 ligation; the ensuing down-regulation of IL-12 restricts the IFN-γ production by T cells thus reducing the size of the response (Berg et al., 2006; Ebrahimkhani et al., 2011). The anatomical location of the LSECs and KCs in the sinusoidal lumen makes them excellent scavengers of a plethora of exogenous antigens and enables them to present these efficiently in the context of MHC I and II molecules *in vivo* (Limmer et al., 2000) for the induction of tolerance. The tolerizing effects of anti-inflammatory responses of liver APCs is typically observed *in vivo* to low levels of LPS. Both human and rodent LSECs upregulate MHC I and express MHC II molecules in response, for example, to viral and bacterial infections or exposure of the liver to IFN-γ (Steinhoff et al., 1988; Steiniger et al., 1988; Steinhoff, 1990). Similar to KCs and classical splenic DCs, LSECs can cross-present hepatocyte-associated antigens to CD8 T cells (Berg et al., 2006; Ebrahimkhani et al., 2011). In contrast, several functional populations of DCs, the professional APCs, are located in the periportal connective tissue of the liver lobule (Sumpter et al., 2007), and they respond to virus infection by elaborating type I IFN, which ultimately leads to the induction of a pro-inflammatory environment in the liver. Apart from the most abundant plasmacytoid DCs, the mouse liver also harbors myeloid DCs and conventional CD8α^+^DCs; thus far, the cCD8α^+^DCs have no counterpart in the human liver. Amongst the three liver DC subsets, the cCD8α^+^DCs have the highest APC capacity.

## The Hepatic Cycle of *Plasmodium*—Implications for Antigen Presentation in the Liver

Following *Plasmodium* infection, mammalian hosts are exposed to *Plasmodium* antigens in multiple ways: (1) sporozoite surface antigens released from viable migrating parasites, (2) sporozoite antigens from parasites that degrade or are eliminated by phagocytosis before they reach their final destination in the liver, (3) early or late LS antigens from parasites that fail to complete the hepatic development cycle, and (4) early or late LS antigens, and potentially blood stage antigens, left behind in the remains of dead infected hepatocytes after successful merosome release.

It has been widely accepted that responses to pre-erythrocytic stage antigens are rather low in persons residing in *P. falciparum* endemic areas (Doolan and Martinez-Alier, 2006). The current model suggests amongst others, that the few invading sporozoites leave inadequate antigen levels or that the sporozoites or the LS antigens that are invisible to the host undergo incomplete or faulty antigen processing for presentation to T cells. Consequently, induction of protective T cell and antibody responses by the erythrocytic stage antigens is rarely observed in naturally infected persons. It is also possible that the pre-erythrocytic antigens themselves induce some regulatory T cells or tolerance, both of which can dampen potential protective responses (Espinoza Mora Mdel et al., 2014; Wilson et al., 2015). Immunization with *P. falciparum* sporozoites, however, can induce high levels of sporozoite-neutralizing antibodies (Nardin, 2010) thus emphasizing the relevance of studying the presentation of sporozoite antigens such as circumsporozoite protein (CSP). Protective immunity requires CD8 memory T cells and CD4 helper T cell-derived cytokines such as IFN-γ that elicit sporozoite-neutralizing antibodies and inhibit LS development mainly through upregulation of iNOS and induction of NO in the infected hepatocytes (Doolan and Martinez-Alier, 2006; Nardin, 2010; Dups et al., 2014). Clinical trials conducted during the past decades revealed that synthetic CSP-derived peptide vaccines induce CD4 and CD8 T cells of similar fine specificity and function (Calvo-Calle et al., 2006; Oliveira et al., 2008; Othoro et al., 2009; Nardin, 2010). Another sporozoite surface antigen, the thrombospondin-related adhesive protein (TRAP), induces transient cytokine responses under natural exposure conditions (Moormann et al., 2009) and central memory T cell responses against TRAP are associated with a significantly reduced incidence of malaria (Todryk et al., 2008). These findings support recent vaccination strategies aimed at inducing durable protective T cell responses against TRAP (Ewer et al., 2013). Interestingly, it was shown that long-term residents of hyperendemic areas in Africa, who received protracted daily anti-malarial prophylactic chloroquine treatment to prevent the development of blood stage infection, developed a strong antibody response to sporozoites and LSs, but only a weak response to blood stages (Marchand and Druilhe, 1990; Gruner et al., 2003). In fact, sera from the individuals displaying responses to sporozoites and immunity against LS were used successfully to identify two LS-specific antigens in *P. falciparum* (Guerin-Marchand et al., 1987; Sangani et al., 1990). A similar example of T cell responses to pre-erythrocytic stage antigens comes from the *P. vivax* malaria. A recently published study shows that persons, who are negative for the Duffy antigen and hence are refractory to *P. vivax* blood-stage infection, do develop stronger T cell responses to sporozoite and LS antigens than Duffy antigen-positive subjects (Wang et al., 2005). These studies suggest that responses to the pre-erythrocytic stage antigens, be it those associated with sporozoites or with LS, are indeed inducible. As suggested by others, the presence of blood stage infection, which vastly exceeds the antigenic load of the sporozoites and the LS, may negatively influence the induction of T and B cell responses. Inhibition of antigen presentation by blood stage antigens has indeed been amply demonstrated in human (Urban et al., 2005) and animal (Ocana-Morgner et al., 2003) *Plasmodium* infections. In the following, we discuss how the main liver cell types could be involved in the acquisition and presentation of different *Plasmodium* antigens.

### Sinusoidal Endothelia

**FIGURE 2:**
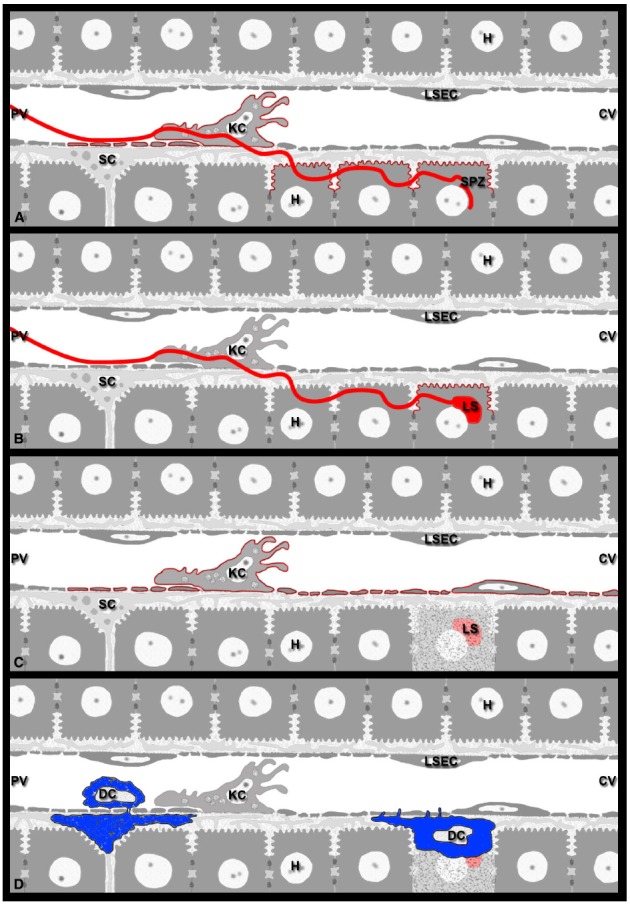
**Model for *Plasmodium* antigen presentation in the liver. (A)** Sporozoites continuously release antigens such as CSP and TRAP from their surface. On their way from the sinusoidal lumen into the liver parenchyma, sporozoites glide along LSECs, traverse KCs, and migrate through several hepatocytes before infecting a final one (red line). In naïve mice, contact-mediated translocation of sporozoite antigens into the cytoplasm of these liver cells may result in antigen presentation by LSECs, KCs, as well as traversed and infected hepatocytes (red outline). **(B)** Sporozoite infection of naïve mice should allow late-LS antigen expression exclusively on infected hepatocytes, i.e., those cells in which the parasites develop (red outline). **(C)** During repeated exposure to viable sporozoites or multiple rounds of immunization with attenuated parasites, infected hepatocytes die after completion of LS development, resulting in the release of debris and leftover late-LS antigens into the environment. These late-LS antigens may then be internalized by nearby APCs such as LSECs and KCs and cross-presented to CD8 effector T cells monitoring the liver. **(D)** Of the various DC subsets that are involved in inflammatory processes of the liver, the conventional CD8α^+^DCs are required for the CD8 T cell-mediated elimination of *Plasmodium* LS. While their exact location in the liver is unknown, *Plasmodium* infection likely attracts immature cCD8α^+^DCs to the hepatic sinusoids, where they interact with KCs and acquire sporozoite antigens. Extravasation into the space of Disse would allow these DCs (blue) to communicate with CD8 effector T cells patrolling the sinusoids. DCs may also internalize cellular and parasite debris released from dead infected hepatocytes and subsequently cross-present these antigens to CD8 effector T cells, either from within the space of Disse or after traveling to the LNs that drain the liver.

*In vitro* studies revealed that during parasite invasion and traversal, *Plasmodium* sporozoites continuously release surface antigens such as CSP and TRAP as part of gliding motility (Stewart and Vanderberg, 1988, 1991, 1992; Hügel et al., 1996; Spaccapelo et al., 1997). In addition, sporozoites can also translocate CSP into the cytosol of mammalian cells by mere membrane contact (Hügel et al., 1996; Frevert et al., 1998; Pradel and Frevert, 2001). The implications for antigen processing and presentation of this phenomenon, which occurs in the absence of invasion, are discussed below. Various cell types of the liver are likely exposed to sporozoite-released antigens. As sporozoites glide extensively along the sinusoids before infecting hepatocytes (Frevert et al., 2005), a substantial number of LSECs are likely exposed to released CSP (Figure 2), which they can present for a recall of both CD8 and CD4 effector T cells (Knolle and Gerken, 2000; Crispe, 2011). LSECs have also been established as the primary cross-presenting APCs of the liver (Limmer et al., 2005). However, cross-presentation by LSECs can also lead to CD8 T cell tolerance (Berg et al., 2006). Thus, while LSECs are candidate APCs, their actual contribution to protective immunity against *Plasmodium* CSP or other sporozoite-associated antigens has not been fully investigated and remains rather difficult to predict.

### Kupffer Cells

The vast majority of sporozoite entry events into the hepatic parenchyma involve KCs (Baer et al., 2007b; Tavares et al., 2013), resident macrophages and professional APCs of the liver (Crispe, 2011; Figure 2). Early evidence supporting this notion came from electron micrographs showing *P. berghei* sporozoites that stretch from the sinusoidal lumen through KCs all the way into hepatocytes (Meis and Verhave, 1988). Subsequently, *P. yoelii* sporozoites were shown to recognize KC-specific surface proteoglycans (Pradel et al., 2002, 2004) and to pass through KCs on their way into the hepatocytes (Frevert et al., 2005). The use of KC-deficient mouse models initially implied an obligatory role of these hepatic macrophages for liver infection (Baer et al., 2007b). A more recent *in vivo* study confirmed the involvement of KCs in the vast majority of all cell traversal events (Tavares et al., 2013), but also demonstrated that some intravenously injected sporozoites use a paracellular pathway for liver infection (Tavares et al., 2013). Thus, while there is no absolute requirement for KC passage, most sporozoite traversal events involve KCs, either directly or indirectly.

Based on the finding that the vast majority of SPECT^–/–^ *P. berghei* sporozoites were trapped in lasting interactions with KCs, it was proposed that sporozoites must use cell traversal to avoid clearance by KC in the liver (Tavares et al., 2013). Of note, once intracellular, these mutant parasites cannot egress from the KC, and therefore die. This is in contrast to viable WT *Plasmodium* sporozoites, which are not killed by KCs, or other macrophages, from naïve mice (Pradel and Frevert, 2001; Frevert et al., 2006; Klotz and Frevert, 2008). Further, the infectious dose for naïve mice is often less than three intravenously injected *P. yoelii* sporozoites (Conteh et al., 2010). Thus, sporozoite infection of the liver is highly efficient, which renders the possibility unlikely that KCs from naïve mice kill sporozoites *in vivo*. On the other hand, 10% of intravenously injected WT *P. berghei* sporozoites were reported to be degraded during KC traversal (Tavares et al., 2013). This disparity could be due to the different parasite species used for the two studies. Alternatively, as the viability of isolated sporozoite preparations varies, some of the *P. berghei* parasites might have been already dead when they arrived in the liver resulting in their clearance by phagocytosis. In general, all available evidence suggests that viable WT sporozoites survive the interaction with naïve KCs unharmed.

In a separate study assessing the *in vivo* responses of KCs to radiation-attenuated *P. berghei* sporozoites (Pb-RAS), infectious sporozoites were shown to down-modulate MHC I and IL-12p40 expression (Steers et al., 2005). However, infectious sporozoite challenge of mice previously immunized with Pb-RAS has the opposite effect in that the expression of MHC I, co-stimulatory molecules, and IL-12 are upregulated. In addition, *in vitro* assessed APC function of these KCs was significantly enhanced in relation to KCs obtained from naïve mice, naïve mice exposed to infectious sporozoites or even mice immunized thrice with Pb-RAS (Steers et al., 2005). Thus, although the prevailing state of immune-tolerance in the liver might attract *Plasmodium* sporozoites to invade hepatocytes where they remain “incognito” as they expand in number, induction of an inflammatory milieu by Pb-RAS reverses the hospitality of the liver to a state of immunologic conflagration that is needed to eliminate the *Plasmodium* parasite. Exposure of mice to doses of IL-12 has a similar, albeit short-lived effect on *Plasmodium* LS (Sedegah et al., 1994; Hoffman et al., 1997). By contrast, Pb-RAS immunization induces lasting protection that becomes refractory to multiple challenges. The reason why KCs respond differentially to RAS versus viable sporozoites is unknown to date. Immunization with the similarly protective genetically attenuated sporozoites (GAS, Khan et al., 2012; Nganou-Makamdop and Sauerwein, 2013) or chemically attenuated sporozoites (CAS, Purcell et al., 2008) may provide insight into the response of KCs, or other hepatic APCs, to sporozoite encounters that are required for protection. Further, careful monitoring of location and fate of the various attenuated parasites may also provide valuable information.

Thus, the hepatic microenvironment from naïve and immune animals is functionally distinct. While KCs from naïve mice become deactivated upon contact with viable sporozoites and eventually succumb to apoptosis (Steers et al., 2005; Klotz and Frevert, 2008), activated KCs may phagocytose sporozoites, in particular if immunization has generated high antibody levels. As in other hepatic diseases (Nagy, 2003), the pro-inflammatory microenvironment created by activated KCs could further enhance the elimination of subsequent cohorts of sporozoites in a local fashion. Depending on the number of attenuated sporozoites used for immunization, challenge with viable sporozoites may allow a few parasites to enter the liver. The finding that challenge of immune mice with viable sporozoites resulted in a very low in number of very small LS supports this idea (Cabrera et al., 2013). In conclusion, the argument that sporozoites must avoid KC contact to successfully infect the liver (Tavares et al., 2013) could potentially be valid for the immune, but not for the naive host.

Whether KCs act as APCs to recall effector T cells in the *Plasmodium* infected liver is unknown to date (reviewed in Frevert, 2004; Frevert et al., 2006, 2008b, 2014; Frevert and Nardin, 2008). Owing to the unique immunological properties of the liver (Knolle and Limmer, 2001; Sheth and Bankey, 2001; Bertolino et al., 2002; Mackay, 2002; Racanelli and Rehermann, 2006; Crispe, 2009), KCs may respond to sporozoite contact with tolerance rather than inflammation and immunity (Frevert et al., 2006). *P. berghei* and *P. yoelii* sporozoites enter KCs actively by formation of a vacuole and release CSP into the cytosol of these APCs *in vitro* (Pradel and Frevert, 2001), suggesting that KCs can present sporozoite surface antigens to both CD8 and CD4 effector T cells (Figure 2). However, a number of reports support the notion that KCs and other macrophages do not survive sporozoite contact (Danforth et al., 1980; Smith and Alexander, 1986; Vanderberg et al., 1990; Klotz and Frevert, 2008). *In vitro* studies revealed that *P. yoelii* sporozoite CSP binds to the low density lipoprotein receptor-related protein (LRP) and proteoglycans on the surface of KCs, which leads to blockage of NADPH oxidase activation, suppression of the respiratory burst, and generation of an anti-inflammatory cytokine secretion profile (Usynin et al., 2007; Klotz and Frevert, 2008). Thus, traversed KCs may not be able to present sporozoite antigens, because they lose APC function and succumb to apoptosis (Klotz and Frevert, 2008). This notion is supported by data from other experimental systems, in which KCs generate tolerance by inducing apoptosis in naïve CD8 T cells in the absence of inflammation (Holz et al., 2012).

Another important finding was that sporozoites are able to introduce CSP into mammalian cells without invading them (Frevert et al., 1996; Hügel et al., 1996). CSP translocation across the cell membrane into the cytosol requires neither the metabolic nor the endocytic machinery of the affected cell (Hügel et al., 1996; Frevert et al., 1998; Pradel and Frevert, 2001) suggesting that it involves a PEXEL motif (Singh et al., 2007). Once in the cytosol, CSP binds to ribosomal RNA (Hügel et al., 1996), which interferes with initiation step of protein synthesis (Frevert, 1999). In contrast to the large number of enzymatically active cytotoxic plant, fungal, and bacterial proteins, which require only a few molecules in the cytosol to kill a cell (Stirpe et al., 1992; Barbieri et al., 1993; Lacadena et al., 2007), CSP has a stoichiometric mode of action (Frevert et al., 1998; Frevert, 1999). The effective concentration of CSP leading to complete inhibition of translation likely varies with the cell type, but complete blockage of the protein synthesis machinery renders uninfected mammalian cells moribund (Frevert et al., 1998). For example, large and metabolically highly active cells such as hepatocytes, which are filled with a huge amount of free and rough endoplasmic reticulum-associated ribosomes, are necessarily less sensitive to the stoichiometric ribotoxic effect of CSP than the much smaller LSECs and KC with a much less developed protein synthesis machinery. In agreement with this notion, macrophages appeared to be particularly sensitive to the ribotoxic action of CSP (Frevert et al., 1998). Nothing is known about the possibility that sporozoite-mediated CSP translocation induces apoptosis in LSECs and KCs. The resulting loss of these APCs from the normally continuous sinusoidal cell layer would expose the underlying parenchyma at the site of sporozoite gliding and KC traversal, similar to the gaps generated by clodronate-mediated KC removal from the sinusoidal cell layer (Baer et al., 2007b), albeit to a much larger extent. As a consequence, effector T cells might gain direct access to traversed and/or infected hepatocytes.

In conclusion, we are only beginning to understand the contribution of KC to sporozoite infection of the liver and the effector phase of immunity against *Plasmodium* LS. KC may be deactivated or even killed by contact with viable sporozoites or by the presence of cytosolic CSP. Alternatively, KC may be activated by exposure to opsonized sporozoites or immunization with attenuated parasites and turned into a source of pro-inflammatory cytokines. A better understanding of these very different scenarios will reveal whether KC (and LSEC) are able to process and present sporozoite antigens for priming of naïve CD8 and CD4 T cells and for induction of recall responses in effector memory T cells.

### Dendritic Cells

Hepatic DCs are thought to play a major role in the cross-presentation of hepatocyte-derived antigens (Bertolino and Bowen, 2015). Under resting conditions, however, hepatic DCs are immature. Due to their location in the periportal interstitium and draining hepatic LNs, these professional APCs are not directly accessible to CD8 effector T cells patrolling the hepatic sinusoids (Figure 2). Pathological or inflammatory conditions trigger the recruitment of blood-borne DC precursors from the bone marrow to the liver sinusoids (Yoneyama and Ichida, 2005). In a murine model of *Propionibacterium acnes*-induced granuloma model, 6 h were required for a significant number of myeloid DCs and plasmacytoid DC precursors to accumulate in the liver of mice with preexisting granulomata (Yoneyama and Ichida, 2005). After antigen uptake, DCs enter the space of Disse, travel via the portal Glisson’s capsule to the draining LNs for antigen presentation to T cells followed by their activation (Sato et al., 1998). Thus, unlike liver-resident APCs (LSECs and KCs), DCs must first travel to the liver, which takes time. Other immune cells also arrive in the liver with a considerable delay. For example, increased numbers of Ly6C^hi^ monocytes, which enter sites of inflammation and get activated locally, were detectable 1 day after systemic infection with *Listeria monocytogenes* (Shi et al., 2010). Further, continuous intravital monitoring revealed that myelomonocytic cells begin to arrive in and extravasate out of hepatic sinusoids roughly an hour after surgical preparation of the liver for IVM (Frevert et al., 2005). Since leukocyte accumulation occurred independently of sporozoite infection, the recruitment of the cells is interpreted as a response to injury.

With respect to malaria, the relative contribution of liver resident versus recruited inflammatory DC is unknown to date (Mauduit et al., 2012). Several studies have demonstrated a critical role of CD8α^+^DCs in the activation of effector CD8 T cells responding to an epitope of CSP. Mice that are missing CD8α^+^DC succumbed to infection in the aftermath of exposure to infectious sporozoites (Layseca-Espinosa et al., 2013). In a murine model of protective immunity induced by *P. berghei* RAS, CD8α^+^DC accumulated in the liver coincident with the exposure to Pb-RAS sporozoites and induced CD8 T cells *in vitro* to produce IFN-γ, an activity that is IL-12 and MHC I dependent (Jobe et al., 2009). Several possible scenarios have been proposed for the cellular mechanisms by which CD8α^+^DCs contribute to protective immunity (Jobe et al., 2009). CD8α^+^DCs might activate effector CD8 T cells in the portal fields or in the draining hepatic LNs, similar to plasmacytoid DCs, which normally do not remain in the Disse spaces for extended periods of time (Yoneyama and Ichida, 2005). Alternatively, CD8α^+^DCs might be recruited by KCs located in the vicinity of infected hepatocytes and extravasate into the spaces of Disse, similar to what has been observed for myeloid DCs in the *P. acnes* model (Yoneyama and Ichida, 2005). This would provide the advantage that *Plasmodium*-specific CTLs could be activated directly in the sinusoid and that the resulting release of cytokines and chemokines could kill LS at relatively close range. Quantitative aspects are worth considering as well. It could be argued that DC accumulation in the liver may not be necessary for CD8 T cell activation, because *in vitro* studies indicate that one CTL is sufficient to kill infected hepatocytes or *Plasmodium* peptide-primed target cells (Bongfen et al., 2007; Frevert et al., 2008a; Trimnell et al., 2009). However, sterile protection against *Plasmodium* infection requires extreme numbers of CD8 effector T cells in the mouse model (Schmidt et al., 2010) and the recruitment of large numbers of CD8 T cells to the vicinity of LS (Cockburn et al., 2013). Together with the apparent absence of direct CTL contact with the dying infected hepatocytes (Cockburn et al., 2013), these data suggest that parasite elimination is relatively inefficient *in vivo* (see below). Therefore, the recruitment of large numbers of DC to the liver may be required for optimal antigen uptake to assure efficient presentation to and activation of T cells in the draining LN. This unusual situation where antigens are loaded onto APCs (in this case DCs) in the liver for subsequent presentation in another organ, the draining LN, may cause a large fraction of the malaria antigens to undergo extensive proteolysis, resulting in a rather limited repertoire of antigenic peptides to activate T cells. Further, if effector memory CD8 T cells are activated in the draining LN they might return to the liver with a considerable delay to kill LS and prevent the erythrocytic phase of the infection.

In conclusion, a combination of DC recruitment to the liver, subsequent DC migration to the draining LN resulting in a limited peptide repertoire, delayed T cell migration back to the liver, and the impossibility to use direct contact-mediated cytotoxicity (see below) may indeed render the CTL response quite ineffective in conferring sterile protection to the host. Clearly, more work is required to elucidate the relative contribution of recruited versus resident hepatic DCs to the presentation of *Plasmodium* antigens to T cells. Dynamic *in vivo* studies should be particularly suitable to shed light onto the local cellular interactions involved in immunity.

### Hepatocytes

After crossing the sinusoidal cell layer, sporozoites traverse several hepatocytes before selecting a final cell for multiplication and differentiation to thousands of merozoites (Mota et al., 2001; Frevert et al., 2005). As mentioned above, sporozoites continuously release surface antigens while migrating (Stewart and Vanderberg, 1988, 1991, 1992; Spaccapelo et al., 1997). The first microscopic evidence for the presence of sporozoite-released CSP in the cytosol of infected hepatoma cells came from the Frevert lab (Hügel et al., 1996; Frevert et al., 1998). Temporal analysis of infected HepG2 cells revealed that the amount of cytosolic CSP decreases with increasing maturity of the intracellular LS. Further, because only a few of the skin-deposited sporozoites get a chance to infect hepatocytes, the amount of CSP available in the liver for either direct presentation to CD8 T cells or for cross-presentation involving non-parenchymal cells is limited (Chakravarty et al., 2007). Nevertheless, as *Plasmodium* increases its chances for survival in the liver by preventing the death of its host cell (van de Sand et al., 2005; Sturm et al., 2006; Rennenberg et al., 2010; Graewe et al., 2012; Kaushansky et al., 2013), antigen presentation to CD8 T cells might continue until merozoite replication and merosome release are accomplished. Two groups proposed that *Plasmodium* LS antigen presentation to CD8 effector T cells is restricted to hepatocytes. First, by transplanting bone marrow from C57BL/6 donor mice into lethally irradiated BALB/c recipient mice, Chakravarty et al. (2007) created C57BL/6 → BALB/c (H-2K^b^ → H-2K^d^) bone marrow chimera. Adoptive transfer of CS-TCR Tg CD8 T cells (H-2K^d^) into these chimera inhibited LS development, but not in the reverse chimeric mice (Chakravarty et al., 2007). On the basis of these observations, the authors suggested that CD8 effector T cells must recognize CSP-derived antigen on hepatocytes to eliminate *Plasmodium* LS from the liver. However, a role of radio-resistant LSECs and stellate cells (SCs) cannot be excluded, because these cells do not originate from the bone marrow (Bertolino and Bowen, 2015). Further, sessile KCs are radiation-resistant (Klein et al., 2007) and kinetic studies revealed that 85% of the recipient KCs remain in the liver 2 months after standard bone marrow transfer (BMT); even after 1 year, less than half of the original KC population is replaced (Kennedy and Abkowitz, 1997; Seki et al., 2009; Inokuchi et al., 2011). Therefore, to fully reconstitute bone marrow derived cells in the liver, KCs must be depleted with clodronate liposomes prior to irradiation and BMT (Van Rooijen and Sanders, 1996). This strategy could be used to accurately address the question of the role of KCs as APCs for *Plasmodium*-specific effector T cells. In this scenario, KC may activate CD8 effector T cells to release inflammatory cytokines, such as IFN-γ, to eliminate *Plasmodium* parasites within infected hepatocytes. In a separate study involving OT-I cells, it was observed that these T cells significantly inhibited the parasite burden in C57BL/6 mice and bone marrow TAP^–/–^ → C57BL/6 chimeras infected with *P. berghei*-OVA transgenic parasites, but not in C57BL/6 → TAP^–/–^ chimeras and TAP^–/–^ mice infected with the same parasites. Kimura et al. (2013) concluded that infected hepatocytes process and present the OVA epitope via the classical cytosolic MHC I pathway.

Two decades ago, immunoelectron microscopy demonstrated CSP in the cytosol of HepG2 cells harboring *P. berghei* sporozoites (Hügel et al., 1996). In retrospect, we know now that these sporozoites were traversing the hepatoma cells (Mota et al., 2001; Frevert et al., 2005), because they were not enclosed in a parasitophorous vacuole as required for productive infection (Mota et al., 2001). Based on these *in vitro* data, it seems likely that both traversed and infected hepatocytes contain sporozoite surface antigens in the liver (Figure 2). *In vivo* studies, on the other hand, suggest that hepatocytes do not survive sporozoite traversal, but undergo necrosis (Frevert et al., 2005). As a consequence, sporozoite antigens associated with the remains of these disintegrating hepatocytes are likely phagocytosed and could be cross-presented by liver APCs. In contrast, only infected hepatocytes would contain early-late-LS, and eventually merozoite antigens within the PV (Butler et al., 2011). Indeed, *in vitro* studies with primary hepatocytes indicate that both traversed and infected cells are able to process and present antigen to induce IFN-γ secretion in primed CD8 T cells (Bongfen et al., 2007). However, *in vitro* exposure of primary hepatocytes to either WT or cell traversal-deficient Pb-RAS revealed that only infected hepatocytes process and present CSP via the proteasome pathway (Bongfen et al., 2008), which led to the proposal that only infected hepatocytes could induce their own elimination (Balam et al., 2012). Results from *in vivo* experiments in a murine model revealed the capacity of hepatocytes to induce protection by priming naïve T cells and by presenting *Plasmodium* CSP to antigen-specific primed CD8 T cells (Balam et al., 2012). As mice immunized with WT and traversal-deficient sporozoites exhibited similar CD8 T cell responses to CSP, the authors suggested that infected hepatocytes play a dominant role in CD8 T cell priming *in vivo*. This interpretation appears to contrast with the observation that hepatic CD8α^+^DCs play a critical role in the activation of CD8 effector T cells after immunization with Pb-RAS (Jobe et al., 2009). Although it is entirely possible that different cells act as APCs during CD8 T cell priming in response to infection with attenuated sporozoites, these APCs should express MHC-I:peptide complexes, co-stimulatory signals and produce inflammatory cytokines (Zarling et al., 2013). In addition, priming of CSP-specific responses may also depend on the source of peripheral DCs (Chakravarty et al., 2007; Cockburn et al., 2011). Together with the finding that apoptotic infected hepatocytes can provide antigens to hepatic DCs (Leiriao et al., 2005), and the notion that antigen presentation by hepatocytes tends to have a tolerizing effect (Crispe et al., 2006; Bertolino and Bowen, 2015), these data underscore the need for more work to elucidate the cellular events within the complex architecture of the intact liver with its large number of non-parenchymal cells (Racanelli and Rehermann, 2006; Frevert and Nardin, 2008; Crispe, 2011), some of which exhibit greater APC function than hepatocytes (Bertolino and Bowen, 2015).

Several different scenarios can be envisioned for cell-mediated immunity directed against late-LS antigens in the naïve versus the immunized host. For example, first-time infection of a naïve host with WT sporozoites leads to expression of late-LS antigens only in hepatocytes, the only liver cell type that supports parasite growth and differentiation. Consequently, under experimental conditions that involve adoptive transfer of late-LS antigen-specific CD8 CTLs into first-time infected recipient mice, these CTLs should be able to recognize late-LS antigens exclusively on infected hepatocytes (Figure 2). Although most merozoites are successfully released into the blood, some parasite debris and LS remnant bodies are left behind and inflammatory cells infiltrate the exhausted former host cell (Baer et al., 2007a). If some of these late-LS antigens are picked up by liver APC, then CTL responses could be activated that would take effect against subsequent generations of incoming parasites. For example, in mice immunized with parasites under drug cover (Belnoue et al., 2004, 2008; Renia et al., 2013), which have experienced several complete rounds of LS development, late-LS antigens left behind after merosome release are likely internalized and cross-presented by hepatic APCs, in particular LSECs and DCs (Figure 2). Similarly, repeated exposure of humans to infected mosquito bites in endemic areas may cause hepatic APCs to cross-present late-LS antigens. Thus, under natural infection conditions, CTLs patrolling the sinusoids might recognize late-LS antigens on LSECs, DCs, and/or KCs in addition to infected hepatocytes. Finally, hepatic APCs may also present late-LS antigens after vaccination with late-stage arrested GAS. We anticipate that advances in longitudinal imaging with sensitive molecular tools will reveal the time of parasite antigen persistence and the fate of GAS-infected host cells.

In conclusion, a few parasites engage with a minute number of hepatic APC in a highly local fashion within a huge organ. Consequently, the fate of the LSECs and KCs that have been touched or traversed by migrating sporozoites *in vivo* remains unknown to date. We are confident that tools can be developed and strategies designed to identify the few APCs at a time when the parasites have already left, be it by imaging or otherwise, for analysis of phenotypical changes in response to infection and immunization.

## Contact-Dependent Mechanisms against *Plasmodium* Liver Stage Parasites

The mechanism by which effector cells eliminate *Plasmodium* LS from the liver has been a matter of contention for decades (Doolan and Hoffman, 2000). While *Plasmodium*-specific CTLs can induce apoptosis in target cells by formation of an immunological synapse (Frevert et al., 2008a; Trimnell et al., 2009), followed by contact-mediated cytotoxicity, the situation is more complex in the liver, where infected hepatocytes are hidden behind a layer of sinusoidal cells. Histological liver sections, although suggestive (Rodrigues et al., 1992), are inadequate to confirm CD8 T cell contact with infected hepatocytes and granule-mediated cytotoxicity. Electron microscopy offers the resolution to visualize CD8 T cell extravasation into the space of Disse, formation of an immunological synapse, and chromatin condensation indicating apoptotic target cell death (Frevert et al., 2008a). However, comprehensive analysis of the small number of LS developing in the large liver requires a more efficient method. For example, dynamic *in vivo* imaging has been used successfully to visualize subcellular details on the liver phase of *Plasmodium* including sporozoite invasion, merozoite maturation, and merosome release into the blood (Sturm et al., 2006; Baer et al., 2007a). However, identification of the exact position of effector T cells in the hepatic microenvironment by intravital imaging requires simultaneous labeling of several crucial structural elements: the vascular lumen, the sinusoidal endothelium, the space of Disse, hepatocytes, and the intracellular parasites. With the recent introduction of a novel imaging technique, intravital reflection recording, it is now possible to monitor the behavior of T cells in the hepatic microvasculature of non-fluorescent mice (Cabrera and Frevert, 2012). Combined with vascular tracers, cellular or molecular markers, and fluorescent reporter mice, intravital reflection recording represents a powerful technique to define the exact location of effector T cells with respect to infected hepatocytes and neighboring non-parenchymal cells, sinusoidal endothelia and KCs.

Three recent studies aimed to identify the mechanism by which CD8 effector T cells kill *Plasmodium* LS in the liver. Using a 70–90% pure preparation of PyCSP TCR-Tg CD8 T cells specific for the *P. yoelii* CSP epitope SYVPSAEQI, Cockburn et al. (2013) showed that CD8 effector T cells cluster around *Plasmodium* LS in the BALB/c mouse liver signaling via G protein-coupled receptors. Both antigen-specific and antigen-unrelated OT-I cells exhibited the same low velocity inside the cluster. Based on this report, PyCSP TCR-Tg cells were proposed to recruit antigen-unrelated OT-I cells to the site of *P. yoelii* LS development (Bayarsaikhan et al., 2015) supporting the argument that all patrolling CD8 T cells will slow down in an area where adhesion molecules are upregulated, because there is no mechanism for recruitment to be antigen specific, only engagement. Surprisingly, however, both the parasite-specific T cells and the OT-I cells moved at slow speed also outside the cluster (Cockburn et al., 2013). Other experimental systems showed that when non-lymphoid tissue resident memory CD8 T cells encounter cognate antigen, they recruit pre-formed circulating memory CD8 T cells of the same specificity to the site of infection (Schenkel et al., 2013). This specialized feature of tissue resident CD8 T cells allows for an accumulation of a large number of effector CD8 T cells prior to the protracted process re-activation and arrival of antigen-specific cells induced in the secondary lymphoid organs, e.g., the draining LN (Schenkel et al., 2013). Another example suggesting that recruitment of CD8 T cells is strictly antigen-dependent comes from studies of tumor killing by activated CTLs. The tumor antigen-specific CD8 T cells migrate at high velocity (∼10 μm/min) in the periphery of tumors expressing cognate antigen, deeply infiltrate the tumors, and kill tumors in a contact-dependent fashion (Boissonnas et al., 2007). In contrast, the same CTLs do not infiltrate unrelated tumors and neither arrest nor kill tumor cells that fail to present cognate antigen.

One reason for the large size of the PyCSP TCR-Tg CD8 T cell clusters (80 μm diameter) may be that the CD8 T cells received MHCI:SYVPSAEQI signals from sporozoite-traversed cells in addition to sporozoite-infected hepatocytes. This possibility is difficult to evaluate, however, because the exact location of the PyCSP TCR-Tg cells with respect to the sinusoidal walls is not visible in that study (Cockburn et al., 2013). Whether these cells were intra- or extravascular in this experimental setting based on a single CSP-derived peptide remains to be shown. In addition, whether large numbers of effector T cells also accumulate around *Plasmodium* LS in natural infections or after immunization with attenuated sporozoites, in particular in the large human liver, also remains to be shown. Immunization with a viral prime boost regimen against multiple epitopes in the *P. falciparum* TRAP revealed that much lower levels of IFN-γ producing CD8 T cells were required for protection of humans (Ewer et al., 2013) compared to the high CD8 T cell frequencies necessary for protection in mice (Schmidt et al., 2010). According to the authors (Ewer et al., 2013), the significantly longer duration of *P. falciparum* LS development, which lasts about 7 days compared to the 2 days for rodent species, could be a plausible explanation for this difference.

In agreement with the concealed location of hepatocytes in the liver, CD8 T cells appear to use different mechanisms for *Plasmodium* LS elimination *in vitro* and *in vivo*. Similar to classical granule-mediated target cell killing, which is typically triggered within seconds of CTL conjugation (Berke, 1995; Keefe et al., 2005; Pipkin and Lieberman, 2007), *P. yoelii* specific CD8 T cells rapidly induce apoptosis in infected hepatocytes *in vitro* and kill the parasites efficiently using a contact- and perforin-dependent, but IFN-γ independent mechanism (Trimnell et al., 2009). In contrast, *P. yoelii* LS killing *in vivo* is a protracted process that is contact-independent and require multiple CD8 T cells per parasite (Cockburn et al., 2013). Together with the finding that CD8 T cells from *P. yoelii* GAS-immunized mice rely partly on IFN-γ for protection (Trimnell et al., 2009), these intravital observations suggest that cytokines are responsible for the PyCSP TCR-Tg cell-mediated LS killing (Cockburn et al., 2013). Compared to classical granule-mediated cytotoxicity, cytokine-mediated effector mechanisms operate slower and, depending on the local cytokine concentration, may range from mere growth stagnation to parasite death.

Another study showed that OT-I CD8 T cells accumulate in very large numbers around mature LS in the livers of naïve C57BL/6 mice 40–48 h after infection with *P. berghei*-gfpOVA (Kimura et al., 2013). Whether or not these T cell clusters formed around all LS was not reported, but as LS development is highly asynchronous, some LS had likely matured and released merosomes, which is typically followed by infiltration of the remains of the dead hepatocytes with inflammatory cells (Khan and Vanderberg, 1991a; Baer et al., 2007a). Alternatively, the clusters were formed by T cells specific for late-stage antigens that appear in the hepatocyte cytoplasm, in this case OVA (Bayarsaikhan et al., 2015). Depending on the host-parasite combination, mice can also mount inflammatory responses prior to LS maturation. For example, *P. berghei* sporozoites are 2000-fold less infectious to BALB/c mice than to C57BL/6 mice (Scheller et al., 1994). On the other hand, *P. yoelii* is 50–100 times more infectious to C57BL/6 mice than *P. berghei* (Briones et al., 1996). As a result, the *P. berghei* parasite biomass in the C57BL/6 mouse liver is significantly lower than that of *P. yoelii* suggesting that *P. berghei* induces an inflammatory response not only in BALB/c mice, but also in C57BL/6 mice. Granuloma formation in naïve *P. berghei*-infected mouse livers starts at 24 h after infection and is therefore clearly independent of adaptive immune mechanisms. The finding that *P. yoelii* LS develop basically undetected in the mouse until merosome release is accomplished (Khan and Vanderberg, 1991b; Liehl et al., 2014) supports this notion (see caveat below). Taken together, CD8 effector T cells appear to participate in granuloma formation around moribund *P. berghei*-infected hepatocytes (Kimura et al., 2013). The alternative hypothesis, namely that CD8 T cells cluster around healthy LS that successfully develop in the liver of naïve mice or humans, requires experimental confirmation.

A third study presents novel imaging techniques that proved essential for visualization of the exact location of CD8 effector memory T cells in the *Plasmodium*-infected liver (Cabrera and Frevert, 2012). Intravital reflection recording revealed that immunization of BALB/c mice with radiation-attenuated *P. yoelii* sporozoites (Py-RAS), early-stage genetically attenuated (Py-uis4^–/–^), or late-stage genetically attenuated (Py-fabb/f^–/–^) parasites significantly increased the velocity of CD8 T cells patrolling the hepatic microvasculature compared to naïve mice (Cabrera et al., 2013). After adoptive transfer, CD8 T cells from these immunized donor mice unexpectedly remained immobile in the hepatic microvasculature for at least 3 days, whether or not the recipient mice were infected (Cabrera et al., 2013). The same low velocity was observed after transfer of TCR-tg CD8 T cells specific for the *P. yoelii* CSP_280–288_ epitope (Butler et al., 2010; Schmidt et al., 2011). Instead of migrating with a leading edge and a trailing uropod as in immunized mice, the transferred CD8 T cells were rounded (Cabrera et al., 2013) and met the definition of local confinement (Friedl and Weigelin, 2008). In fact, an 28% pure PyCSP TCR-tg CD8 effector memory T cells exhibited a similarly low average velocity after adoptive transfer as the 70–90% pure PyCSP TCR-Tg CD8 effector T cells mentioned above (Cockburn et al., 2013). Although recipient mice were infected with one million sporozoites, a parasite density that should have facilitated CTL encounters with LS, none of the transferred CD8 T cells, whether of hepatic or splenic origin, approached or made contact with infected hepatocytes under any of the experimental conditions used (Cabrera et al., 2013). Because neither immunized donor mice nor infected recipient mice provided any evidence for CD8 T cell extravasation into the space of Disse, the CD8 effector memory T cells from the immunized mice likely eliminated infected hepatocytes via release of soluble mediators, cytokines and/or chemokines (Frevert and Nacer, 2013; Miller et al., 2014). The finding that adoptive transfer of two million purified CD8 T cells from Pb-uis3^–/–^ immunized mice conferred sterile protection to challenge with 50,000 viable sporozoites supports this notion (Mueller et al., 2007).

In conclusion, while conducted under very different conditions in terms of *Plasmodium* species, CD8 T cell specificity, and time of imaging, common denominators that emerge from these studies are (1) the low velocity of adoptively transferred CD8 T cells, whether antigen-specific or not and whether in proximity to the parasites or not, in the liver of recipient mice, (2) the lack of direct CD8 T cell contact with infected hepatocytes, and (3) the considerably slower LS killing *in vivo* compared to hepatocyte monocultures, where CTLs have direct access infected hepatocytes.

## Adoptive Transfer-Associated Phenotypical CD8 T Cell Changes in Other Systems

Much of our current understanding of T cell functions comes from experiments using adoptive transfers of either enriched T cell subsets displaying a certain phenotype or T cells bearing a TCR-Tg for a given specificity. Recent advances in the use of intravital imaging have provided us with a much clearer understanding of T cell functions vis-à-vis the patterns of migratory behavior within the various lymphoid and non-lymphoid organs. The microarchitecture of each organ and the expression of MHC:peptide complexes, chemokines, adhesion molecules and cytokines provide signaling pathways that prompt specific movement and velocity patterns, as well as changes in T cell function. Collectively, these observations suggest a certain plasticity of most T cells is needed mainly to prevent tissue pathology, while simultaneously enhancing T cell effector function against invading pathogens. For example, liver CD8 T cells specific for HBV modulate their IFN-γ production and cytolytic function in an oscillatory and sequential fashion, which culminates in effective reduction of viral load (Isogawa et al., 2005). Th17 cells convert to IFN-γ producing Th1-like cells after adoptive transfer into NOD/SCID recipient mice (Bending et al., 2009; Martin-Orozco et al., 2009). Further, adoptively transferred resting memory CD8 T cells acquire an effector phenotype upon entry into non-lymphoid tissues as indicated by induction of lytic activity and granzyme B expression (Marzo et al., 2007). Some of these observed changes may simply reflect a programmed T cell differentiation, e.g., from resting memory to effector cells, upon reinfection. Changes in the profiles of cytokine production or lytic function may also indicate that anatomical location plays a role in local T cell differentiation.

## Cytokine-Mediated Effector Mechanisms against *Plasmodium* Liver Stages

Nonspecific responses to microenvironmental changes could provide an alternative explanation for the finding that antibody-mediated blockage of IFN-γ abrogated protection against *Plasmodium* LS within 2 days (Schofield et al., 1987b; Rodrigues et al., 1991; Weiss et al., 1992), but neither 8 days after CD8 T cell transfer (Chakravarty et al., 2008) nor in immunized mice in the absence of adoptive transfer (Doolan and Hoffman, 2000). Whatever the cause of the immobility of adoptively transferred CD8 effector T cells, this phenomenon is useful to demonstrate that direct contact is not necessary for CTL-mediated protection against *Plasmodium* LS. Blockage of IFN-γ within 2 days after transfer abrogates protection (Schofield et al., 1987b; Rodrigues et al., 1991; Weiss et al., 1992), a result that supports the concept that soluble factors are obligatory for protection when CTLs are unable to approach infected hepatocytes. A role of cytokines, in particular IFN-γ and TNF-α, in parasite killing is clearly documented, both *in vivo* and *in vitro* (Ferreira et al., 1986; Mellouk et al., 1987, 1991; Schofield et al., 1987a; Rodrigues et al., 1991; Weiss et al., 1992; Seguin et al., 1994; Renggli et al., 1997; Doolan and Hoffman, 2000; Jobe et al., 2007; Mueller et al., 2007; Butler et al., 2010). For example, C57BL/6 mice deficient in both perforin and the CD95/CD95L pathway were protected after immunization with Pb-RAS suggesting that parasite-specific CD8 effector T cell-derived cytokines activate mechanisms responsible for the elimination of the intracellular LS (Renggli et al., 1997).

IFN-γ is now considered the central mediator of protection against LS (McCall and Sauerwein, 2010). However, the finding that (1) blockage of IFN-γ does not abolish protection in actively immunized mice (Doolan and Hoffman, 2000), (2) CD8 T cells from IFN-γ deficient mice protect mice challenge 8 days after transfer (Chakravarty et al., 2008), when the CTLs have likely regained motility, and (3) CD8 T cells protect IFN-γ KO mice against infection (Butler et al., 2010) all suggest that IFN-γ is not the only soluble factor involved. CD8 T cells monitor hepatocytes with small cytoplasmic projections that reach into the space of Disse (Warren et al., 2006), but they appear not to extravasate into the liver tissue or form immunological synapses with hepatocytes *in vivo* (Crispe, 2011). Interestingly, while perforin-dependent cytotoxicity plays an important role in the clearance of virus infections from extrahepatic organs, this mechanism was not involved in the elimination of hepatocytes infected with a non-cytopathic adenovirus from the liver (Kafrouni et al., 2001). Because hepatic CD8 T cells exhibited similar IFN-γ and TNF responses and were able to kill virus-infected cells *in vitro*, this finding was interpreted as a relative resistance of hepatocytes to perforin-mediated cytotoxicity (Kafrouni et al., 2001). Alternatively, however, CD8 T cells may be unable to use contact-dependent cytotoxicity because they cannot exit the hepatic sinusoid (Cabrera et al., 2013; Frevert and Nacer, 2013). Of note, CTLs can form two different types of immunological synapses (1) lytic synapses, used for target cell killing and (2) stimulatory synapses with APCs to induce cytokine secretion (Faroudi et al., 2003; Depoil et al., 2005; Wiedemann et al., 2006). It seems therefore possible that CTLs specific for *Plasmodium* sporozoite proteins recognize—from within the sinusoidal lumen—antigens at the site of parasite entry into the parenchyma, form stimulatory synapses with sinusoidal APC, in particular KCs, secrete IFN-γ in a multidirectional fashion (Kupfer et al., 1991; Huse et al., 2006; Sanderson et al., 2012), and trigger the secretion of additional cytokines such as IL-6 and IL-12 that then contribute to LS killing (Nüssler et al., 1991; Pied et al., 1991, 1992; Vreden et al., 1992; Belnoue et al., 2004). Synapse formation with sinusoidal APCs could involve cytotoxic granule proteins (Doolan and Hoffman, 2000; Butler et al., 2010), which might explain the requirement for perforin in protection of vaccinated humans (Seder et al., 2013) and mice (Butler et al., 2010). This scenario could also pertain to CTLs that recognize late-LS antigens on cross-presenting APCs.

How can CTL-derived cytokines reach infected hepatocytes (Figure 1)? Considering the high sinusoid-to-lymph filtration rate (Greenway and Lautt, 1970; Laine et al., 1979; Henriksen et al., 1984), the enhanced pressure gradients created by leukocytes moving through the sinusoidal lumen (Wisse et al., 1983), and the highly anastomosed sinusoidal microvasculature, CD8 T cells could conceivably exploit both the anterograde blood flow and the retrograde lymph flow to take control of a large portion of the liver lobule—without extravasation and granule-mediated cytotoxicity (Frevert and Nacer, 2013). This hypothesis is supported by the finding that intravenous IFN-γ inoculation reduced the *P. berghei* liver burden independently of the sporozoite challenge dose (Ferreira et al., 1986). Further, CD8 T cells protect against various viral liver infections and *in vivo* data indicate that virus replication is blocked without CD8 T cell extravasation and in the absence of hepatocyte death (Guidotti et al., 1999; Kafrouni et al., 2001; Guidotti, 2002; Crispe, 2011). Consistent with the notion that CTL-derived cytokines traverse the sieve plates of the sinusoidal endothelia and disseminate via the lymphatic conduits of the liver, adoptively transferred-specific CTLs acted against infected hepatocytes, but had no effect on not infected renal tubule or choroid plexus epithelia (Ando et al., 1994a), both of which are shielded from the bloodstream by a continuous layer of non-fenestrated endothelia.

While the crucial role of perforin expressing CD8 T cells in protection against *Plasmodium* LS is now well documented (Renggli et al., 1997; Doolan and Hoffman, 2000; Trimnell et al., 2009; Butler et al., 2010), the exact mechanism by which this cytotoxic granule protein contributes to the elimination of infected hepatocytes *in vivo* is unclear. CTLs typically release perforin into an immunological synapse (Lieberman, 2003; Pipkin and Lieberman, 2007; Lopez et al., 2013). Unlike Fas/FasL-mediated cytotoxicity, which requires only a few molecules on the target cell and which could therefore potentially be accomplished via small cytoplasmic projections such as TEHLI (Warren et al., 2006), immunological synapse formation involves a much larger area of contact between CTL and target cell (Dustin, 2005; Pipkin and Lieberman, 2007). For this reason, establishment of synapse with the basolateral hepatocyte membrane likely requires extravasation of the CTL. However, CTLs conjugating with hepatocytes from within the space of Disse have not been documented to date, neither in malaria nor in other liver infections. While CTLs specific for the were initially thought to exert a direct cytopathic effect on infected hepatocytes *in vivo* (Ando et al., 1994b), the use of a transgenic mouse model of hepatitis B infection revealed that adoptively transferred virus-specific CTLs abolish gene expression and replication of the virus in the liver in the absence of hepatocyte death (Guidotti and Chisari, 1996; Guidotti et al., 1999; Kafrouni et al., 2001; Wuensch et al., 2006; Giannandrea et al., 2009). Thus, rather than reflecting secretion of cytotoxic molecules and induction of apoptosis, which requires immunological synapse formation with the target cell, the requirement of perforin expression for protection may indicate CD8 T cell maturation in the liver. This model would also link perforin expression to IFN-γ secretion. It is now established that CTLs eliminate the virus by secretion of IFN-γ and TNF-α and that this cytokine-mediated, non-cytopathic mechanism represents a survival strategy of the host to control massive viral infections of vital organs such as the liver (Guidotti, 2002). Clearly, widespread takeover of the hepatocyte machinery by a hepatotropic virus differs from the very focal intracellular development of a large protozoan parasite such as *Plasmodium*. However, all available evidence suggests that both infectious agents can be eliminated via cytokines, i.e., in the absence of CTL extravasation and immunological synapse formation with antigen-presenting hepatocytes. Taken together, CTLs appear to be more likely to engage in stimulatory immunological synapse formation with sinusoidal APCs rather than establishing lytic synapses with infected hepatocytes. However, this aspect of effector CD8 T cell activity will need to be investigated further. Confocal or multi-photon microscopy, combined with cellular and molecular tools for intravital imaging of the hepatic microvasculature (Cabrera and Frevert, 2012), can provide the necessary spatio-temporal resolution to elucidate which liver cell types interact with which *Plasmodium* antigen-specific CD8 T cells and whether discrete epitopes of the still unknown liver-stage specific antigens require specific hepatocyte and/or APC contacts.

Much is known about the cytotoxic mechanisms associated with immunological synapse formation *in vitro* (Russell and Ley, 2002; Catalfamo and Henkart, 2003; Lieberman, 2003; Trambas and Griffiths, 2003). For example, upon *in vitro* conjugation of human-specific CD8 T cells with cognate target cells, perforin rapidly accumulates at the immunological synapse where it promotes cytotoxicity (Makedonas et al., 2009). Although cellular cytotoxicity against congenic hepatocytes has been studied extensively in various virus hepatitis models (Guidotti et al., 1999; Kafrouni et al., 2001; Wuensch et al., 2006; Giannandrea et al., 2009), CTL conjugation with target hepatocytes in the intact liver has not been documented to our knowledge, neither ultrastructurally nor with the combined use of modern fluorescent tools and state-of-the-art imaging techniques. Unlike the continuous endothelium of other organs, which prevents ready access of soluble mediators to the parenchyma, one of the essential functions of the fenestrated sinusoidal endothelium of the liver is to allow unrestricted passage of soluble and small corpuscular substances to hepatocytes for detoxification and a plethora of anabolic and catabolic processes. Further, the liver is unique in that every hepatocyte is in direct contact with sinusoidal endothelia thus allowing screening by patrolling immune cells and exposure to their secreted cytokines. This is in stark contrast to other organs, which require immune cell infiltration for elimination of infected, malignant, or otherwise abnormal target cells. This applies in particular to organs with a tight blood barrier such as brain or testes or tissues comprised of a dense parenchyma with a relatively low degree of vascularization such as joint or cartilage.

In conclusion, it appears that CD8 effector T cells recognize the site of sporozoite entry into the liver, either by detecting antigen presented on hepatic local APC or by screening hepatocytes via TEHLI. Being unable to extravasate and kill via classical granule-mediated cytotoxicity, CTLs appear to eliminate *Plasmodium* LS via secretion of cytokines, in particular IFN-γ.

## Cytokine-Mediated Control of Parasite Growth

Cytokines alone can clearly confer protection against *Plasmodium* LS, further supporting the notion that CTL-mediated cytotoxicity does not require contact with infected hepatocytes. *In vitro* studies document that *P. yoelii* and *P. falciparum* infected hepatocytes can be eliminated by direct exposure to IFN-γ (Ferreira et al., 1986; Mellouk et al., 1991, 1994) and intravenous IFN-γ inoculation reduced liver burden in a *P. berghei* model (Ferreira et al., 1986). Antibodies against CD8 T cells or IFN-γ abrogated protection (Ferreira et al., 1986), suggesting that CD8 T cells either secrete sufficient IFN-γ or produce other cytokines that elicit IFN-γ secretion from other cellular sources (Schofield et al., 1987b). Clearly, IFN-γ mediated protection is dependent on iNOS-mediated production in infected hepatocytes (Mellouk et al., 1991, 1994; Nussler et al., 1993; Seguin et al., 1994; Klotz et al., 1995). However, despite the essential role of IFN-γ in iNOS upregulation in infected hepatocytes (Nussler et al., 1993; Seguin et al., 1994; Klotz et al., 1995), the exact mode of operation of this cytokine *in vivo* is not known (Doolan and Hoffman, 2000; Overstreet et al., 2008; Butler et al., 2010). Interestingly, IFN-γ induced iNOS activation was shown to regulate the replication of various obligatory intracellular microorganisms including *Toxoplasma* (Pfefferkorn, 1984, 1986; Pfefferkorn et al., 1986; Takacs et al., 2012), *Trypanosoma cruzi* (Silva et al., 2003), *Leishmania* (Bogdan et al., 2000), *Mycobacterium* (Herbst et al., 2011), and *Chlamydia* (Zhang et al., 2012). An IFN-γ based regulatory mechanism could explain why challenge of immunized mice results not only in a smaller number of *Plasmodium* LS (as expected for a direct killing mechanism), but also in a reduced LS size (Cabrera et al., 2013). In agreement with this finding, the effect of IFN-γ induced iNOS activation may range from a minor reduction in LS growth to complete cure of the host cell depending on the local CD8 T cell density, the level of IFN-γ secretion, the position of the T cells and the LS relative to the liver lobule, and the distance between them.

Thus, it appears that CTLs exert an IFN-γ mediated growth-regulatory effect on *Plasmodium* LS rather than acting in a parasitocidal fashion. To be efficient, this mechanism requires an organ with sinusoidal endothelia, a large *trans*-endothelial lymph filtration rate, and a high microvascular density that exposes every parenchymal cell to CTL-derived cytokines—properties uniquely combined in the liver. Considering that a similar mechanism of cellular immunity has been proposed to operate against viral infections of the liver (Guidotti, 2002; Guidotti and Iannacone, 2013), IFN-γ induced iNOS activation in hepatocytes (Chen et al., 2003) may have evolved as an efficient strategy to control hepatotropic microbes while promoting hepatocyte survival and preserving the function of this essential organ.

## Contribution of Cellular Effector Mechanisms to Protection of Naïve Versus Immune Hosts

*Plasmodium* has developed multiple parallel strategies to evade detection in the liver: the choice of a tolerogenic environment for its initial round of replication, the limited infection of a minute number of hepatocytes in the huge liver, and the change of protein expression from sporozoite to late-LS antigens. For these reasons, it has been suggested that finding and killing all LS during the brief period of LS development represents the predominant challenge the CD8 T cell response is facing (Bertolino and Bowen, 2015). Indeed, this reasoning clearly applies to the most severe malaria cases, namely to first infections of naïve hosts that result in a fatal outcome after only one round of LS development. In these cases, effective CTL-mediated interruption of LS development would prevent the clinically symptomatic blood infection in young *P. falciparum*-infected children and also allow susceptible mice to survive experimental infection with large numbers of lethal *P. yoelii* XL or *P. berghei* ANKA sporozoites.

After repeated infections, for example with non-lethal parasite species/strains or with sub-lethal inoculation doses, protection increasingly relies on the humoral arm of immunity. Challenge of immunized mice by mosquito bite likely allows antibodies to immobilize most sporozoites in the skin (Vanderberg and Frevert, 2004). Should a few parasites manage to escape from the skin, they are likely opsonized in the bloodstream and phagocytosed by KCs. In contrast to liver infection of naïve mice, which is very fast and highly efficient (Shin et al., 1982; Conteh et al., 2010), studies with mice that had been vaccinated with *P. yoelii* RAS or GAS showed that the majority of intravenously inoculated sporozoites are unable to enter the liver (Cabrera et al., 2013). Thus, LS development in the immune host is impeded and extremely scarce, which drastically reduces the probability of parasite recognition by *Plasmodium*-specific CD8 T cells, in particular in the large human liver. These considerations emphasize the crucial role of antibodies in protection of the previously exposed host against recurring sporozoite infections (Vanderberg et al., 2007; Vanderberg, 2014).

## Contribution of Innate Immune Responses to Protection against *Plasmodium* LS

Recent studies demonstrate a significant impact of the innate immune response on the survival of *Plasmodium* in the murine liver. Both *P. berghei* and *P. yoelii* infected hepatocytes were shown to sense *Plasmodium* LS and induce a type I IFN response that is propagated by hepatocytes in an interferon-a/b receptor dependent fashion and reduces the parasite burden in the liver (Liehl et al., 2014; Miller et al., 2014). In the *P. yoelii* model, primary infection with a late-stage GAP increased the number of IFN-γ secreting CD1d-restricted NKT cells in the liver thus implicating IFN-γ as a crucial innate factor in controlling secondary infection with WT XNL parasites (Miller et al., 2014). Although lymphocytes were required for the innate suppression of secondary liver infections, CD8 T cells, CD4 T cells, and NK cells, the latter of which constituted the largest subset of IFN-γ secreting cells, appeared to be dispensable as effector cells. Type I IFN signaling was responsible for the recruitment of both CD8 T cells and CD49b^+^CD3^+^ NKT cells to the liver, but only NKT cells reduced the LS burden significantly (Miller et al., 2014), which is in line with earlier work (Gonzalez-Aseguinolaza et al., 2000).

Certain differences between the two murine models are noteworthy. For example, the innate immune response to *P. berghei* infection involved the cytosolic pattern recognition receptor MDA5, the adaptor molecule for cytosolic RNA sensors, MAVS, suggesting parasite RNA sensing, and the transcription factors IRF3 and IRF7 (Liehl et al., 2014). In the *P. yoelii* model, IRF3, but not IRF7, was crucial for the induction of the type I IFN response and neither MDA5 nor MAVS contributed to a functional innate response against secondary infection (Miller et al., 2014). Further, *P. berghei* infection activated a considerably stronger innate immune response compared to *P. yoelii*, and interferon-stimulated gene expression was upregulated at 36 h after infection with WT *P. berghei*, but only at 42 h after infection with WT *P. yoelii* (Liehl et al., 2014). In agreement with earlier histological work (Khan and Vanderberg, 1991b), immune cell infiltration occurred midway through completion of LS development for *P. berghei*, but only at the time of merosome formation for *P. yoelii* (Miller et al., 2014). Finally, IFN-γ signaling pathways were upregulated as early as 24 h after infection with late-stage arrested *P. yoelii* parasites (Miller et al., 2014), while early stage-arrested *P. berghei* parasites (GAS or RAS) failed to induce such a response (Liehl et al., 2014).

Thus, many questions remain. For example, is the intense inflammation and early granuloma formation associated with *P. berghei* infection of the murine liver the cause or the consequence of the type I IFN response? Is parasite death required for RNA sensing and initiation of the innate response? Can LS sensing be improved by parasite attenuation? Addressing these issues may provide crucial clues as to which events in the life cycle reveal the presence of the parasite to the host. Perhaps most relevant for malaria endemic areas: does the human host indeed sense intracellular LS if infected with a well adapted *Plasmodium* species?

## Outlook and Future Directions

We hope to have highlighted the vast number of open questions associated with the local cellular events leading to protection against *Plasmodium* LS. While much progress has been made on the biology and nutritional requirements of *Plasmodium* LS (Luder et al., 2009; Vaughan et al., 2009; Rankin et al., 2010; Labaied et al., 2011; Deschermeier et al., 2012; Graewe et al., 2012; Itoe et al., 2014), the liver is still a black box in terms of effector mechanisms mediating the detection and elimination of infected hepatocytes. Recent live attenuated malaria vaccination trials in humans and non-human primates as well as work in mice clearly demonstrate the requirement for protection of large quantities of IFN-γ producing CD8, CD4, and γδ T cells as well as IFN-γ secreting NK and NKT cells in the liver (Epstein et al., 2011; Teirlinck et al., 2011; Seder et al., 2013). Neither location nor behavior in the liver of any of these cells nor their exact modes of action during adaptive and innate immunity has been determined, however. Elucidation of the protective cell-mediated mechanisms elicited by attenuated sporozoite vaccines will aid in the development of a cheap, safe, and easy-to-administer synthetic vaccine that matches this gold standard. As hundreds of millions of people living in endemic areas are in desperate need for protection against malaria, future vaccination protocols should be selected based on their capacity to boost the number of CD8 T cells. Indeed, a multi-epitope malaria vaccine that significantly increases the magnitude of T cell induction has already been developed (Ewer et al., 2013). This so-called ME-TRAP vaccine contains full-length *Plasmodium falciparum* TRAP fused to ME, a string of 20 malarial T and B cell epitopes (McConkey et al., 2003).

### Conflict of Interest Statement

The authors declare that the research was conducted in the absence of any commercial or financial relationships that could be construed as a potential conflict of interest.

## Abbreviations

APC: antigen-presenting cell
BM: bone marrow
BMT: bone marrow transfer
CSP: circumsporozoite protein
DC: dendritic cell
GAS: genetically attenuated sporozoites
KC: Kupffer cell
LSEC: liver sinusoidal endothelial cell
RAS: radiation-attenuated sporozoites
TCR-Tg: T cell receptor transgenic.

## References

[B1] Ando K. Guidotti L. G. Cerny A. Ishikawa T. Chisari F. V. (1994a). CTL access to tissue antigen is restricted in vivo. J. Immunol. 153, 482–488. 8021489

[B2] Ando K. Guidotti L. G. Wirth S. Ishikawa T. Missale G. Moriyama T. (1994b). Class I-restricted cytotoxic T lymphocytes are directly cytopathic for their target cells in vivo. J. Immunol. 152, 3245–3253. 8144915

[B3] Baer K. Klotz C. Kappe S. H. Schnieder T. Frevert U. (2007a). Release of hepatic *Plasmodium yoelii* merozoites into the pulmonary microvasculature. PLoS Pathog. 3:e171. 10.1371/journal.ppat.0030171 17997605 PMC2065874

[B4] Baer K. Roosevelt M. Van Rooijen N. Clarkson A. B. Jr. Schnieder T. (2007b). Kupffer cells are obligatory for *Plasmodium yoelii* sporozoite infection of the liver. Cell. Microbiol. 9, 397–412. 10.1111/j.1462-5822.2006.00798.x 16953803

[B5] Balam S. Romero J. F. Bongfen S. E. Guillaume P. Corradin G. (2012). CSP-A model for in vivo presentation of *Plasmodium berghei* sporozoite antigens by hepatocytes. PLoS ONE 7:e51875. 10.1371/journal.pone.0051875 23272182 PMC3525584

[B6] Barbieri L. Battelli M. G. Stirpe F. (1993). Ribosome-inactivating proteins from plants. Biochim. Biophys. Acta 1154, 237–282. 10.1016/0304-4157(93)90002-6 8280743

[B7] Bayarsaikhan G. Akbari M. Yui K. Amino K. (2015). Antigen-driven focal inflammatory death of malaria liver stages. Front. Microbiol. 6:47. 10.3389/fmicb.2015.00047 25699034 PMC4316770

[B8] Belnoue E. Costa F. T. Frankenberg T. Vigario A. M. Voza T. Leroy N. (2004). Protective T cell immunity against malaria liver stage after vaccination with live sporozoites under chloroquine treatment. J. Immunol. 172, 2487–2495. 10.4049/jimmunol.172.4.2487 14764721

[B9] Belnoue E. Voza T. Costa F. T. Gruner A. C. Mauduit M. Rosa D. S. (2008). Vaccination with live *Plasmodium yoelii* blood stage parasites under chloroquine cover induces cross-stage immunity against malaria liver stage. J. Immunol. 181, 8552–8558. 10.4049/jimmunol.181.12.8552 19050274 PMC5551044

[B10] Bending D. De La Pena H. Veldhoen M. Phillips J. M. Uyttenhove C. Stockinger B. (2009). Highly purified Th17 cells from BDC2.5NOD mice convert into Th1-like cells in NOD/SCID recipient mice. J. Clin. Invest. 119, 565–572. 10.1172/JCI37865 19188681 PMC2648686

[B11] Berg M. Wingender G. Djandji D. Hegenbarth S. Momburg F. Hammerling G. (2006). Cross-presentation of antigens from apoptotic tumor cells by liver sinusoidal endothelial cells leads to tumor-specific CD8^+^ T cell tolerance. Eur. J. Immunol. 36, 2960–2970. 10.1002/eji.200636033 17039564

[B12] Berke G. (1995). The CTL’s kiss of death. Cell 81, 9–12. 10.1016/0092-8674(95)90365-8 7536631

[B13] Bertolino P. Bowen D. G. (2015). Malaria and the liver: immunological hide-and-seek or subversion of immunity from within? Front. Microbiol. 6:41. 10.3389/fmicb.2015.00041 25741320 PMC4332352

[B14] Bertolino P. Mccaughan G. W. Bowen D. G. (2002). Role of primary intrahepatic T-cell activation in the ‘liver tolerance effect’. Immunol. Cell Biol. 80, 84–92. 10.1046/j.0818-9641.2001.01048.x 11869365

[B15] Bogdan C. Rollinghoff M. Diefenbach A. (2000). The role of nitric oxide in innate immunity. Immunol. Rev. 173, 17–26. 10.1034/j.1600-065X.2000.917307.x 10719664

[B16] Boissonnas A. Fetler L. Zeelenberg I. S. Hugues S. Amigorena S. (2007). In vivo imaging of cytotoxic T cell infiltration and elimination of a solid tumor. J. Exp. Med. 204, 345–356. 10.1084/jem.20061890 17261634 PMC2118741

[B17] Bongfen S. E. Balam S. Torgler R. Romero J. F. Corradin G. (2008). Processing of the circumsporozoite protein in infected hepatocytes is not dependent on aspartic proteases. Parasite Immunol. 30, 375–378. 10.1111/j.1365-3024.2008.01032.x 18444957

[B18] Bongfen S. E. Torgler R. Romero J. F. Renia L. Corradin G. (2007). *Plasmodium berghei*-infected primary hepatocytes process and present the circumsporozoite protein to specific CD8^+^ T cells in vitro. J. Immunol. 178, 7054–7063. 10.4049/jimmunol.178.11.7054 17513754

[B19] Briones M. R. S. Tsuji M. Nussenzweig V. (1996). The large difference in infectivity for mice of *Plasmodium berghei* and *Plasmodium yoelii* sporozoites cannot be correlated with their ability to enter hepatocytes. Mol. Biochem. Parasitol. 77, 7–17. 10.1016/0166-6851(96)02574-1 8784767

[B20] Burgio V. L. Ballardini G. Artini M. Caratozzolo M. Bianchi F. B. Levrero M. (1998). Expression of co-stimulatory molecules by Kupffer cells in chronic hepatitis of hepatitis C virus etiology. Hepatology 27, 1600–1606. 10.1002/hep.510270620 9620333

[B21] Butler N. S. Schmidt N. W. Harty J. T. (2010). Differential effector pathways regulate memory CD8 T cell immunity against *Plasmodium berghei* versus *P. yoelii* sporozoites. J. Immunol. 184, 2528–2538. 10.4049/jimmunol.0903529 20097864 PMC2904689

[B22] Butler N. S. Schmidt N. W. Vaughan A. M. Aly A. S. Kappe S. H. Harty J. T. (2011). Superior antimalarial immunity after vaccination with late liver stage-arresting genetically attenuated parasites. Cell Host Microbe 9, 451–462. 10.1016/j.chom.2011.05.008 21669394 PMC3117254

[B23] Cabrera M. Frevert U. (2012). Novel in vivo imaging techniques for the liver microvasculature. Intravital 1, 107–114. 10.4161/intv.23423

[B24] Cabrera M. Pewe L. L. Harty J. T. Frevert U. (2013). *In vivo* CD8^+^ T cell dynamics in the liver of *Plasmodium yoelii* immunized and infected mice. PLoS ONE 8: e70842. 10.1371/journal.pone.0070842 23967119 PMC3743839

[B25] Calvo-Calle J. M. Oliveira G. A. Watta C. O. Soverow J. Parra-Lopez C. Nardin E. H. (2006). A linear peptide containing minimal T- and B-cell epitopes of *Plasmodium falciparum* circumsporozoite protein elicits protection against transgenic sporozoite challenge. Infect. Immun. 74, 6929–6939. 10.1128/IAI.01151-06 17030584 PMC1698101

[B26] Catalfamo M. Henkart P. A. (2003). Perforin and the granule exocytosis cytotoxicity pathway. Curr. Opin. Immunol. 15, 522–527. 10.1016/S0952-7915(03)00114-6 14499260

[B27] Chakravarty S. Baldeviano G. C. Overstreet M. G. Zavala F. (2008). Effector CD8^+^ T lymphocytes against liver stages of *Plasmodium yoelii* do not require gamma interferon for antiparasite activity. Infect. Immun. 76, 3628–3631. 10.1128/IAI.00471-08 18519561 PMC2493192

[B28] Chakravarty S. Cockburn I. A. Kuk S. Overstreet M. G. Sacci J. B. Zavala F. (2007). CD8^+^ T lymphocytes protective against malaria liver stages are primed in skin-draining lymph nodes. Nat. Med. 13, 1035–1041. 10.1038/nm1628 17704784

[B29] Chen T. Zamora R. Zuckerbraun B. Billiar T. R. (2003). Role of nitric oxide in liver injury. Curr. Mol. Med. 3, 519–526. 10.2174/1566524033479582 14527083

[B30] Cockburn I. A. Amino R. Kelemen R. K. Kuo S. C. Tse S. W. Radtke A. (2013). In vivo imaging of CD8^+^ T cell-mediated elimination of malaria liver stages. Proc. Natl. Acad. Sci. U.S.A. 110, 9090–9095. 10.1073/pnas.1303858110 23674673 PMC3670364

[B31] Cockburn I. A. Tse S. W. Radtke A. J. Srinivasan P. Chen Y. C. Sinnis P. (2011). Dendritic cells and hepatocytes use distinct pathways to process protective antigen from *Plasmodium in vivo*. PLoS Pathog. 7:e1001318. 10.1371/journal.ppat.1001318 21445239 PMC3060173

[B32] Conteh S. Chattopadhyay R. Anderson C. Hoffman S. L. (2010). *Plasmodium yoelii* -Infected *A. stephensi* inefficiently transmit malaria compared to intravenous route. PLoS ONE 5: e8947. 10.1371/journal.pone.0008947 20126610 PMC2812485

[B33] Crispe I. N. (2009). The liver as a lymphoid organ. Annu. Rev. Immunol. 27, 147–163. 10.1146/annurev.immunol.021908.132629 19302037

[B34] Crispe I. N. (2011). Liver antigen-presenting cells. J. Hepatol. 54, 357–365. 10.1016/j.jhep.2010.10.005 21084131 PMC3031082

[B35] Crispe I. N. (2014). APC licensing and CD4+T cell help in liver-stage malaria. Front. Microbiol. 5:617. 10.3389/fmicb.2014.00617 25426113 PMC4227505

[B36] Crispe I. N. Giannandrea M. Klein I. John B. Sampson B. Wuensch S. (2006). Cellular and molecular mechanisms of liver tolerance. Immunol. Rev. 213, 101–118. 10.1111/j.1600-065X.2006.00435.x 16972899

[B37] Danforth H. D. Aikawa M. Cochrane A. H. Nussenzweig R. S. (1980). Sporozoites of mammalian malaria: attachment to, interiorization and fate within macrophages. J. Protozool. 27, 193–202. 10.1111/j.1550-7408.1980.tb04680.x 6772771

[B38] Depoil D. Zaru R. Guiraud M. Chauveau A. Harriague J. Bismuth G. (2005). Immunological synapses are versatile structures enabling selective T cell polarization. Immunity 22, 185–194. 10.1016/j.immuni.2004.12.010 15723807

[B39] Deschermeier C. Hecht L. S. Bach F. Rutzel K. Stanway R. R. Nagel A. (2012). Mitochondrial lipoic acid scavenging is essential for *Plasmodium berghei* liver stage development. Cell. Microbiol. 14, 416–430. 10.1111/j.1462-5822.2011.01729.x 22128915

[B40] Doolan D. L. Hoffman S. L. (2000). The complexity of protective immunity against liver-stage malaria. J. Immunol. 165, 1453–1462. 10.4049/jimmunol.165.3.1453 10903750

[B41] Doolan D. L. Martinez-Alier N. (2006). Immune response to pre-erythrocytic stages of malaria parasites. Curr. Mol. Med. 6, 169–185. 10.2174/156652406776055249 16515509

[B42] Dups J. N. Pepper M. Cockburn I. A. (2014). Antibody and B cell responses to *Plasmodium* sporozoites. Front. Microbiol. 5:625. 10.3389/fmicb.2014.00625 25477870 PMC4235289

[B43] Dustin M. L. (2005). A dynamic view of the immunological synapse. Semin. Immunol. 17, 400–410. 10.1016/j.smim.2005.09.002 16266811

[B44] Ebrahimkhani M. R. Mohar I. Crispe I. N. (2011). Cross-presentation of antigen by diverse subsets of murine liver cells. Hepatology 54, 1379–1387. 10.1002/hep.24508 21721032 PMC3444257

[B45] Epstein J. E. Tewari K. Lyke K. E. Sim B. K. Billingsley P. F. Laurens M. B. (2011). Live attenuated malaria vaccine designed to protect through hepatic CD8^+^ T cell immunity. Science 334, 475–480. 10.1126/science.1211548 21903775

[B46] Espinoza Mora Mdel R. Steeg C. Tartz S. Heussler V. Sparwasser T. Link A. (2014). Depletion of regulatory T cells augments a vaccine-induced T effector cell response against the liver-stage of malaria but fails to increase memory. PLoS ONE 9:e104627. 10.1371/journal.pone.0104627 25115805 PMC4130546

[B47] Ewer K. J. O’Hara G. A. Duncan C. J. Collins K. A. Sheehy S. H. Reyes-Sandoval A. (2013). Protective CD8^+^ T-cell immunity to human malaria induced by chimpanzee adenovirus-MVA immunisation. Nat. Commun. 4, 2836. 10.1038/ncomms3836 24284865 PMC3868203

[B48] Faroudi M. Utzny C. Salio M. Cerundolo V. Guiraud M. Muller S. (2003). Lytic versus stimulatory synapse in cytotoxic T lymphocyte/target cell interaction: manifestation of a dual activation threshold. Proc. Natl. Acad. Sci. U.S.A. 100, 14145–14150. 10.1073/pnas.2334336100 14610278 PMC283560

[B49] Ferreira A. Schofield L. Enea V. Schellekens H. Van Der Meide P. Collins W. E. (1986). Inhibition of development of exoerythrocytic forms of malaria parasites by gamma-interferon. Science 232, 881–884. 10.1126/science.3085218 3085218

[B50] Frevert U. (1999). Heparan sulphate and RNA-binding sites in the malaria circumsporozoite protein. Biochem. Soc. Trans. 27, 482–487. 10917626 10.1042/bst0270482

[B51] Frevert U. (2004). Sneaking in through the back entrance: the biology of malaria liver stages. Trends Parasitol. 20, 417–424. 10.1016/j.pt.2004.07.007 15324732

[B52] Frevert U. Engelmann S. Zougbédé S. Stange J. Ng B. Matuschewski K. (2005). Intravital observation of *Plasmodium berghei* sporozoite infection of the liver. PLoS Biol. 3:e192. 15901208 10.1371/journal.pbio.0030192 PMC1135295

[B53] Frevert U. Galinski M. R. Hügel F.-U. Allon N. Schreier H. Smulevitch S. (1998). Malaria circumsporozoite protein inhibits protein synthesis in mammalian cells. EMBO J. 17, 3816–3826. 10.1093/emboj/17.14.3816 9669999 PMC1170717

[B54] Frevert U. Moreno A. Calvo Calle J. M. Klotz C. Nardin E. (2008a). Imaging human cytotoxic CD4^+^ T cells specific for *Plasmodium falciparum* circumsporozoite protein. Int. J. Parasitol. 39, 119–132. 10.1016/j.ijpara.2008.06.014 18723023 PMC3021960

[B55] Frevert U. Usynin I. Baer K. Klotz C. (2008b). *Plasmodium* sporozoite passage across the sinusoidal cell layer. Subcell. Biochem. 47, 182–197. 10.1007/978-0-387-78267-6_15 18512352

[B56] Frevert U. Nacer A. (2013). Immunobiology of *Plasmodium* in liver and brain. Parasite Immunol. 35, 267–282. 10.1111/pim.12039 23631610

[B57] Frevert U. Nacer A. Cabrera M. Movila A. Leberl M. (2014). Imaging *Plasmodium* immunobiology in the liver, brain, and lung. Parasitol. Int. 63, 171–186. 10.1016/j.parint.2013.09.013 24076429 PMC3876283

[B58] Frevert U. Nardin E. (2008). Cellular effector mechanisms against *Plasmodium* liver stages. Cell. Microbiol. 10, 1956–1967. 10.1111/j.1462-5822.2008.01211.x 18647171

[B59] Frevert U. Sinnis P. Esko J. D. Nussenzweig V. (1996). Cell surface glycosaminoglycans are not obligatory for *Plasmodium berghei* invasion in vitro. Mol. Biochem. Parasitol. 76, 257–266. 10.1016/0166-6851(95)02563-4 8920011

[B60] Frevert U. Usynin I. Baer K. Klotz C. (2006). Nomadic or sessile: can Kupffer cells function as portals for malaria sporozoites to the liver? Cell. Microbiol. 8, 1537–1546. 10.1111/j.1462-5822.2006.00777.x 16911567

[B61] Friedl P. Weigelin B. (2008). Interstitial leukocyte migration and immune function. Nat. Immunol. 9, 960–969. 10.1038/ni.f.212 18711433

[B62] Giannandrea M. Pierce R. H. Crispe I. N. (2009). Indirect action of tumor necrosis factor-alpha in liver injury during the CD8+ T cell response to an adeno-associated virus vector in mice. Hepatology 49, 2010–2020. 10.1002/hep.22869 19291774 PMC2871665

[B63] Gonzalez-Aseguinolaza G. De Oliveira C. Tomaska M. Hong S. Bruna-Romero O. Nakayama T. (2000). α-galactosylceramide-activated Vα14 natural killer T cells mediate protection against murine malaria. Proc. Natl. Acad. Sci. U.S.A. 97, 8461–8466. 10.1073/pnas.97.15.8461 10900007 PMC26970

[B64] Graewe S. Stanway R. R. Rennenberg A. Heussler V. T. (2012). Chronicle of a death foretold: *Plasmodium* liver stage parasites decide on the fate of the host cell. FEMS Microbiol. Rev. 36, 111–130. 10.1111/j.1574-6976.2011.00297.x 22092244

[B65] Greenway C. V. Lautt W. W. (1970). Effects of hepatic venous pressure on transsinusoidal fluid transfer in the liver of the anesthetized cat. Circ. Res. 26, 697–703. 10.1161/01.RES.26.6.697 5422930

[B66] Groux H. Bigler M. De Vries J. E. Roncarolo M. G. (1996). Interleukin-10 induces a long-term antigen-specific anergic state in human CD4^+^ T cells. J. Exp. Med. 184, 19–29. 10.1084/jem.184.1.19 8691133 PMC2192687

[B67] Gruner A. C. Snounou G. Brahimi K. Letourneur F. Renia L. Druilhe P. (2003). Pre-erythrocytic antigens of *Plasmodium falciparum*: from rags to riches? Trends Parasitol. 19, 74–78. 10.1016/S1471-4922(02)00067-3 12586475

[B68] Guerin-Marchand C. Druilhe P. Galey B. Londono A. Patarapotikul J. Beaudoin R. L. (1987). A liver-stage-specific antigen of *Plasmodium falciparum* characterized by gene cloning. Nature 329, 164–167. 10.1038/329164a0 3306406

[B69] Guidotti L. G. (2002). The role of cytotoxic T cells and cytokines in the control of hepatitis B virus infection. Vaccine 20(Suppl. 4), A80–A82. 10.1016/S0264-410X(02)00392-4 12477433

[B70] Guidotti L. G. Chisari F. V. (1996). To kill or to cure: options in host defense against viral infection. Curr. Opin. Immunol. 8, 478–483. 10.1016/S0952-7915(96)80034-3 8794011

[B71] Guidotti L. G. Iannacone M. (2013). Effector CD8 T cell trafficking within the liver. Mol. Immunol. 55, 94–99. 10.1016/j.molimm.2012.10.032 23149103 PMC3578146

[B72] Guidotti L. G. Rochford R. Chung J. Shapiro M. Purcell R. Chisari F. V. (1999). Viral clearance without destruction of infected cells during acute HBV infection. Science 284, 825–829. 10.1126/science.284.5415.825 10221919

[B73] Henriksen J. H. Horn T. Christoffersen P. (1984). The blood-lymph barrier in the liver. A review based on morphological and functional concepts of normal and cirrhotic liver. Liver 4, 221–232. 10.1111/j.1600-0676.1984.tb00932.x 6384715

[B74] Herbst S. Schaible U. E. Schneider B. E. (2011). Interferon gamma activated macrophages kill mycobacteria by nitric oxide induced apoptosis. PLoS ONE 6:e19105. 10.1371/journal.pone.0019105 21559306 PMC3085516

[B75] Hoffman S. L. Crutcher J. M. Puri S. K. Ansari A. A. Finkelman F. Gately M. K. (1997). Sterile protection of monkeys against malaria after administration of interleukin-12. Nat. Med. 3, 80–83. 10.1038/nm0197-80 8986746

[B76] Holz L. E. Benseler V. Vo M. Mcguffog C. Van Rooijen N. Mccaughan G. W. (2012). Naive CD8 T cell activation by liver bone marrow-derived cells leads to a “neglected” IL-2^low^ Bim^high^ phenotype, poor CTL function and cell death. J. Hepatol. 57, 830–836. 10.1016/j.jhep.2012.05.015 22659099

[B77] Hügel F.-U. Pradel G. Frevert U. (1996). Release of malaria circumsporozoite protein into the host cell cytoplasm and interaction with ribosomes. Mol. Biochem. Parasitol. 81, 151–170. 10.1016/0166-6851(96)02701-6 8898331

[B78] Huse M. Lillemeier B. F. Kuhns M. S. Chen D. S. Davis M. M. (2006). T cells use two directionally distinct pathways for cytokine secretion. Nat. Immunol. 7, 247–255. 10.1038/ni1304 16444260

[B79] Inokuchi S. Tsukamoto H. Park E. Liu Z. X. Brenner D. A. Seki E. (2011). Toll-like receptor 4 mediates alcohol-induced steatohepatitis through bone marrow-derived and endogenous liver cells in mice. Alcohol. Clin. Exp. Res. 35, 1509–1518. 10.1111/j.1530-0277.2011.01487.x 21463341 PMC3131439

[B80] Isogawa M. Furuichi Y. Chisari F. V. (2005). Oscillating CD8^+^ T cell effector functions after antigen recognition in the liver. Immunity 23, 53–63. 10.1016/j.immuni.2005.05.005 16039579

[B81] Itoe M. A. Sampaio J. L. Cabal G. G. Real E. Zuzarte-Luis V. March S. (2014). Host cell phosphatidylcholine is a key mediator of malaria parasite survival during liver stage infection. Cell Host Microbe 16, 778–786. 10.1016/j.chom.2014.11.006 25498345 PMC4271766

[B82] Jenne C. N. Kubes P. (2013). Immune surveillance by the liver. Nat. Immunol. 14, 996–1006. 10.1038/ni.2691 24048121

[B83] Jobe O. Donofrio G. Sun G. Liepinsh D. Schwenk R. Krzych U. (2009). Immunization with radiation-attenuated *Plasmodium berghei* sporozoites induces liver cCD8α^+^DC that activate CD8^+^T cells against liver-stage malaria. PLoS ONE 4:e5075. 10.1371/journal.pone.0005075 19347042 PMC2661143

[B84] Jobe O. Lumsden J. Mueller A. K. Williams J. Silva-Rivera H. Kappe S. H. (2007). Genetically attenuated *Plasmodium berghei* liver stages induce sterile protracted protection that is mediated by major histocompatibility complex Class I-dependent interferon-γ-producing CD8+ T cells. J. Infect. Dis. 196, 599–607. 10.1086/519743 17624847 PMC3594113

[B85] Kafrouni M. I. Brown G. R. Thiele D. L. (2001). Virally infected hepatocytes are resistant to perforin-dependent CTL effector mechanisms. J. Immunol. 167, 1566–1574. 10.4049/jimmunol.167.3.1566 11466378

[B86] Kaushansky A. Ye A. S. Austin L. S. Mikolajczak S. A. Vaughan A. M. Camargo N. (2013). Suppression of host p53 is critical for *Plasmodium* liver-stage infection. Cell Rep. 3, 630–637. 10.1016/j.celrep.2013.02.010 23478020 PMC3619000

[B87] Keefe D. Shi L. Feske S. Massol R. Navarro F. Kirchhausen T. (2005). Perforin triggers a plasma membrane-repair response that facilitates CTL induction of apoptosis. Immunity 23, 249–262. 10.1016/j.immuni.2005.08.001 16169498

[B88] Kennedy D. W. Abkowitz J. L. (1997). Kinetics of central nervous system microglial and macrophage engraftment: analysis using a transgenic bone marrow transplantation model. Blood 90, 986–993. 9242527

[B89] Khan S. M. Janse C. J. Kappe S. H. Mikolajczak S. A. (2012). Genetic engineering of attenuated malaria parasites for vaccination. Curr. Opin. Biotechnol. 23, 1–9. 10.1016/j.copbio.2012.04.003 22560204

[B90] Khan Z. M. Vanderberg J. P. (1991a). Eosinophil-rich, granulomatous inflammatory response to *Plasmodium berghei* hepatic schizonts in non-immunized rats is age-related. Am. J. Trop. Med. Hyg. 45, 190–201. 1877714 10.4269/ajtmh.1991.45.190

[B91] Khan Z. M. Vanderberg J. P. (1991b). Role of host cellular response in differential susceptibility of nonimmunized BALB/C mice to *Plasmodium berghei* and *Plasmodium yoelii* sporozoites. Infect. Immun. 59, 2529–2534. 1855974 10.1128/iai.59.8.2529-2534.1991 PMC258051

[B92] Kimura K. Kimura D. Matsushima Y. Miyakoda M. Honma K. Yuda M. (2013). CD8^+^ T cells specific for a malaria cytoplasmic antigen form clusters around infected hepatocytes and are protective at the liver stage of infection. Infect Immun. 81, 3825–3834. 10.1128/IAI.00570-13 23897612 PMC3811763

[B93] Klein I. Cornejo J. C. Polakos N. K. John B. Wuensch S. A. Topham D. J. (2007). Kupffer cell heterogeneity: functional properties of bone marrow derived and sessile hepatic macrophages. Blood 110, 4077–4085. 10.1182/blood-2007-02-073841 17690256 PMC2190614

[B94] Klotz C. Frevert U. (2008). *Plasmodium yoelii* sporozoites modulate cytokine profile and induce apoptosis in murine Kupffer cells. Int. J. Parasitol. 38, 1639–1650. 10.1016/j.ijpara.2008.05.018 18656478 PMC2659474

[B95] Klotz F. W. Scheller L. F. Seguin M. C. Kumar N. Marletta M. A. Green S. J. (1995). Co-localization of inducible-nitric oxide synthase and *Plasmodium berghei* in hepatocytes from rats immunized with irradiated sporozoites. J. Immunol. 154, 3391–3395. 7534796

[B96] Knolle P. A. Gerken G. (2000). Local control of the immune response in the liver. Immunol. Rev. 174, 21–34. 10.1034/j.1600-0528.2002.017408.x 10807504

[B97] Knolle P. A. Limmer A. (2001). Neighborhood politics: the immunoregulatory function of organ-resident liver endothelial cells. Trends Immunol. 22, 432–437. 10.1016/S1471-4906(01)01957-3 11473832

[B98] Knolle P. A. Schlaak J. Uhrig A. Kempf P. Meyer Zum Büschenfelde K.-H. Gerken G. (1995). Human Kupffer cells secrete IL-10 in response to lipopolysaccharide (LPS) challenge. J. Hepatol. 22, 226–229. 10.1016/0168-8278(95)80433-1 7790711

[B99] Kupfer A. Mosmann T. R. Kupfer H. (1991). Polarized expression of cytokines in cell conjugates of helper T cells and splenic B cells. Proc. Natl. Acad. Sci. U.S.A. 88, 775–779. 10.1073/pnas.88.3.775 1825141 PMC50896

[B100] Labaied M. Jayabalasingham B. Bano N. Cha S. J. Sandoval J. Guan G. (2011). *Plasmodium* salvages cholesterol internalized by LDL and synthesized *de novo* in the liver. Cell. Microbiol. 13, 569–586. 10.1111/j.1462-5822.2010.01555.x 21105984

[B101] Lacadena J. Alvarez-Garcia E. Carreras-Sangra N. Herrero-Galan E. Alegre-Cebollada J. Garcia-Ortega L. (2007). Fungal ribotoxins: molecular dissection of a family of natural killers. FEMS Microbiol. Rev. 31, 212–237. 10.1111/j.1574-6976.2006.00063.x 17253975

[B102] Laine G. A. Hall J. T. Laine S. H. Granger J. (1979). Transsinusoidal fluid dynamics in canine liver during venous hypertension. Circ. Res. 45, 317–323. 10.1161/01.RES.45.3.317 572270

[B103] Layseca-Espinosa E. Korniotis S. Montandon R. Gras C. Bouillie M. Gonzalez-Amaro R. (2013). CCL22-producing CD8α^–^ myeloid dendritic cells mediate regulatory T cell recruitment in response to G-CSF treatment. J. Immunol. 191, 2266–2272. 10.4049/jimmunol.1202307 23878314

[B104] Leiriao P. Mota M. M. Rodriguez A. (2005). Apoptotic *Plasmodium*-infected hepatocytes provide antigens to liver dendritic cells. J. Infect. Dis. 191, 1576–1581. 10.1086/429635 15838783

[B105] Lieberman J. (2003). The ABCs of granule-mediated cytotoxicity: new weapons in the arsenal. Nat. Rev. Immunol. 3, 361–370. 10.1038/nri1083 12766758

[B106] Liehl P. Zuzarte-Luis V. Chan J. Zillinger T. Baptista F. Carapau D. (2014). Host-cell sensors for *Plasmodium* activate innate immunity against liver-stage infection. Nat. Med. 20, 47–53. 10.1038/nm.3424 24362933 PMC4096771

[B107] Limmer A. Ohl J. Kurts C. Ljunggren H. G. Reiss Y. Groettrup M. (2000). Efficient presentation of exogenous antigen by liver endothelial cells to CD8+ T cells results in antigen-specific T-cell tolerance. Nat. Med. 6, 1348–1354. 10.1038/82161 11100119

[B108] Limmer A. Ohl J. Wingender G. Berg M. Jungerkes F. Schumak B. (2005). Cross-presentation of oral antigens by liver sinusoidal endothelial cells leads to CD8 T cell tolerance. Eur. J. Immunol. 35, 2970–2981. 10.1002/eji.200526034 16163670

[B109] Lopez J. A. Susanto O. Jenkins M. R. Lukoyanova N. Sutton V. R. Law R. H. (2013). Perforin forms transient pores on the target cell plasma membrane to facilitate rapid access of granzymes during killer cell attack. Blood 121, 2659–2668. 10.1182/blood-2012-07-446146 23377437

[B110] Luder C. G. Stanway R. R. Chaussepied M. Langsley G. Heussler V. T. (2009). Intracellular survival of apicomplexan parasites and host cell modification. Int. J. Parasitol. 39, 163–173. 10.1016/j.ijpara.2008.09.013 19000910

[B111] Mackay I. R. (2002). Hepatoimmunology: a perspective. Immunol. Cell Biol. 80, 36–44. 10.1046/j.1440-1711.2002.01063.x 11869361

[B112] Magari S. (1990). Hepatic lymphatic system: structure and function. J. Gastroenterol. Hepatol. 5, 82–93. 10.1111/j.1440-1746.1990.tb01769.x 2103385

[B113] Makedonas G. Banerjee P. P. Pandey R. Hersperger A. R. Sanborn K. B. Hardy G. A. (2009). Rapid up-regulation and granule-independent transport of perforin to the immunological synapse define a novel mechanism of antigen-specific CD8^+^ T cell cytotoxic activity. J. Immunol. 182, 5560–5569. 10.4049/jimmunol.0803945 19380804 PMC2714586

[B114] Marchand C. Druilhe P. (1990). How to select *Plasmodium falciparum* pre-erythrocytic antigens in an expression library without defined probe. Bull. World Health Organ. 68 (Suppl.), 158–164. 1709833 PMC2393044

[B115] Martin-Orozco N. Chung Y. Chang S. H. Wang Y. H. Dong C. (2009). Th17 cells promote pancreatic inflammation but only induce diabetes efficiently in lymphopenic hosts after conversion into Th1 cells. Eur. J. Immunol. 39, 216–224. 10.1002/eji.200838475 19130584 PMC2755057

[B116] Marzo A. L. Yagita H. Lefrancois L. (2007). Cutting edge: migration to nonlymphoid tissues results in functional conversion of central to effector memory CD8 T cells. J. Immunol. 179, 36–40. 10.4049/jimmunol.179.1.36 17579018 PMC2861291

[B117] Mauduit M. See P. Peng K. Renia L. Ginhoux F. (2012). Dendritic cells and the malaria pre-erythrocytic stage. Immunol. Res. 53, 115–126. 10.1007/s12026-012-8269-7 22418726

[B118] McCall M. B. Sauerwein R. W. (2010). Interferon-γ—central mediator of protective immune responses against the pre-erythrocytic and blood stage of malaria. J. Leukoc. Biol. 88, 1131–1143. 10.1189/jlb.0310137 20610802

[B119] McConkey S. J. Reece W. H. Moorthy V. S. Webster D. Dunachie S. Butcher G. (2003). Enhanced T-cell immunogenicity of plasmid DNA vaccines boosted by recombinant modified vaccinia virus Ankara in humans. Nat. Med. 9, 729–735. 10.1038/nm881 12766765

[B120] Meis J. F. Verhave J. P. (1988). Exoerythrocytic development of malarial parasites. Adv. Parasitol. 27, 1–61. 10.1016/S0065-308X(08)60352-8 3289327

[B121] Mellouk S. Green S. J. Nacy C. A. Hoffman S. L. (1991). IFN-γ inhibits development of *Plasmodium berghei* exoerythrocytic stages in hepatocytes by an L-arginine-dependent effector mechanism. J. Immunol. 146, 3971–3976. 1903415

[B122] Mellouk S. Hoffman S. L. Liu Z. Z. De La Vega P. Billiar T. R. Nussler A. K. (1994). Nitric oxide-mediated antiplasmodial activity in human and murine hepatocytes induced by gamma interferon and the parasite itself: enhancement by exogenous tetrahydrobiopterin. Infect. Immun. 62, 4043–4046. 8063424 10.1128/iai.62.9.4043-4046.1994 PMC303065

[B123] Mellouk S. Maheshwari R. K. Rhodes-Feuilette A. Beaudoin R. L. Berbibuier N. Matile H. (1987). Inhibitory activity of interferons and interleukin 1 on the development of *Plasmodium falciparum* in human hepatocyte cultures. J. Immunol. 139, 4192–4195. 2447163

[B124] Miller J. L. Sack B. K. Baldwin M. Vaughan A. M. Kappe S. H. (2014). Interferon-mediated innate immune responses against malaria parasite liver stages. Cell Rep. 7, 436–447. 10.1016/j.celrep.2014.03.018 24703850

[B125] Moormann A. M. Sumba P. O. Tisch D. J. Embury P. King C. H. Kazura J. W. (2009). Stability of interferon-gamma and interleukin-10 responses to *Plasmodium falciparum* liver stage antigen 1 and thrombospondin-related adhesive protein immunodominant epitopes in a highland population from Western Kenya. Am. J. Trop. Med. Hyg. 81, 489–495. 19706920 PMC3634720

[B126] Mota M. M. Pradel G. Vanderberg J. P. Hafalla J. C. Frevert U. Nussenzweig R. S. (2001). Migration of *Plasmodium* sporozoites through cells before infection. Science 291, 141–144. 10.1126/science.291.5501.141 11141568

[B127] Mueller A. K. Deckert M. Heiss K. Goetz K. Matuschewski K. Schluter D. (2007). Genetically attenuated *Plasmodium berghei* liver stages persist and elicit sterile protection primarily via CD8 T cells. Am. J. Pathol. 171, 107–115. 10.2353/ajpath.2007.060792 17591958 PMC1941586

[B128] Nagy L. E. (2003). Recent insights into the role of the innate immune system in the development of alcoholic liver disease. Exp. Biol. Med. (Maywood) 228, 882–890. 12968059 10.1177/153537020322800803

[B129] Nardin E. (2010). The past decade in malaria synthetic peptide vaccine clinical trials. Hum. Vaccin. 6, 27–38. 20173408 10.4161/hv.6.1.9601

[B130] Nganou-Makamdop K. Sauerwein R. W. (2013). Liver or blood-stage arrest during malaria sporozoite immunization: the later the better? Trends Parasitol. 10.1016/j.pt.2013.03.008 [Epub ahead of print]. 23608185

[B131] Nüssler A. Pied S. Goma J. Rénia L. Miltgen F. Grau G. E. (1991). TNF inhibits malaria hepatic stages in vitro via synthesis of IL-6. Int. Immunol. 3, 317–321. 10.1093/intimm/3.4.317 1878339

[B132] Nussler A. K. Renia L. Pasquetto V. Miltgen F. Matile H. Mazier D. (1993). In vivo induction of the nitric oxide pathway in hepatocytes after injection with irradiated malaria sporozoites, malaria blood parasites or adjuvants. Eur. J. Immunol. 23, 882–887. 10.1002/eji.1830230417 8458376

[B133] Ocana-Morgner C. Mota M. M. Rodriguez A. (2003). Malaria blood stage suppression of liver stage immunity by dendritic cells. J. Exp. Med. 197, 143–151. 10.1084/jem.20021072 12538654 PMC2193811

[B134] Ohtani O. Ohtani Y. (2008). Lymph circulation in the liver. Anat. Rec. (Hoboken) 291, 643–652. 10.1002/ar.20681 18484610

[B135] Oliveira G. A. Kumar K. A. Calvo-Calle J. M. Othoro C. Altszuler D. Nussenzweig V. (2008). Class II-restricted protective immunity induced by malaria sporozoites. Infect. Immun. 76, 1200–1206. 10.1128/IAI.00566-07 18160479 PMC2258813

[B136] Othoro C. Johnston D. Lee R. Soverow J. Bystryn J.-C. Nardin E. (2009). Enhanced immunogenicity of *Plasmodium falciparum* peptide vaccines using a topical adjuvant containing a potent synthetic Toll-like receptor 7 agonist, imiquimod. Infect. Immun. 77, 739–748. 10.1128/IAI.00974-08 19047411 PMC2632023

[B137] Overstreet M. G. Cockburn I. A. Chen Y. C. Zavala F. (2008). Protective CD8 T cells against *Plasmodium* liver stages: immunobiology of an ‘unnatural’ immune response. Immunol. Rev. 225, 272–283. 10.1111/j.1600-065X.2008.00671.x 18837788 PMC2597001

[B138] Pfefferkorn E. R. (1984). Interferon gamma blocks the growth of *Toxoplasma gondii* in human fibroblasts by inducing the host cells to degrade tryptophan. Proc. Natl. Acad. Sci. U.S.A. 81, 908–912. 10.1073/pnas.81.3.908 6422465 PMC344948

[B139] Pfefferkorn E. R. (1986). Interferon gamma and the growth of *Toxoplasma gondii* in fibroblasts. Ann. Inst. Pasteur. Microbiol. 137A, 348–352. 10.1016/S0769-2609(86)80047-3 3122643

[B140] Pfefferkorn E. R. Eckel M. Rebhun S. (1986). Interferon-gamma suppresses the growth of *Toxoplasma gondii* in human fibroblasts through starvation for tryptophan. Mol. Biochem. Parasitol. 20, 215–224. 10.1016/0166-6851(86)90101-5 3093859

[B141] Pied S. Civas A. Berlot-Picard F. Rénia L. Miltgen F. Gentilini M. (1992). IL-6 induced by IL-1 inhibits malaria pre-erythrocytic stages but its secretion is down-regulated by the parasite. J. Immunol. 146, 197–201. 1727866

[B142] Pied S. Rénia L. Nüssler A. Miltgen F. Mazier D. (1991). Inhibitory activity of IL-6 on malaria hepatic stages. Parasite Immunol. 13, 211–217. 10.1111/j.1365-3024.1991.tb00276.x 2052407

[B143] Pipkin M. E. Lieberman J. (2007). Delivering the kiss of death: progress on understanding how perforin works. Curr. Opin. Immunol. 19, 301–308. 10.1016/j.coi.2007.04.011 17433871 PMC11484871

[B144] Pradel G. Frevert U. (2001). *Plasmodium* sporozoites actively enter and pass through Kupffer cells prior to hepatocyte invasion. Hepatology 33, 1154–1165. 10.1053/jhep.2001.24237 11343244

[B145] Pradel G. Garapaty S. Frevert U. (2002). Proteoglycans mediate malaria sporozoite targeting to the liver. Mol. Microbiol. 45, 637–651. 10.1046/j.1365-2958.2002.03057.x 12139612

[B146] Pradel G. Garapaty S. Frevert U. (2004). Kupffer and stellate cell proteoglycans mediate malaria sporozoite targeting to the liver. Comp. Hepatol. 3 (Suppl. 1), S47. 10.1046/j.1365-2958.2002.03057.x 14960199 PMC2410262

[B147] Purcell L. A. Wong K. A. Yanow S. K. Lee M. Spithill T. W. Rodriguez A. (2008). Chemically attenuated *Plasmodium* sporozoites induce specific immune responses, sterile immunity and cross-protection against heterologous challenge. Vaccine 26, 4880–4884. 10.1016/j.vaccine.2008.07.017 18672017 PMC2560175

[B148] Racanelli V. Rehermann B. (2006). The liver as an immunological organ. Hepatology 43, S54–S62. 10.1002/hep.21060 16447271

[B149] Radtke A. J. Kastenmuller W. Espinosa D. A. Gerner M. Y. Tse S. W. Sinnis P. (2015). Lymph-node resident CD8α+ dendritic cells capture antigens from migratory malaria sporozoites and induce CD8^+^ T cell responses. PLoS Pathog. 11:e1004637. 10.1371/journal.ppat.1004637 25658939 PMC4450069

[B150] Rankin K. E. Graewe S. Heussler V. T. Stanway R. R. (2010). Imaging liver-stage malaria parasites. Cell. Microbiol. 12, 569–579. 10.1111/j.1462-5822.2010.01454.x 20180802

[B151] Reid L. M. Fiorino A. S. Sigal S. H. Brill S. Holst P. A. (1992). Extracellular matrix gradients in the space of Disse: relevance to liver biology. Hepatology 15, 1198–1203. 10.1002/hep.1840150635 1592356

[B152] Renggli J. Hahne M. Matile H. Betschart B. Tschopp J. Corradin G. (1997). Elimination of *P. berghei* liver stages is independent of Fas (CD95/Apo-I) or perforin-mediated cytotoxicity. Parasite Immunol. 19, 145–148. 10.1046/j.1365-3024.1997.d01-190.x 9106820

[B153] Renia L. Gruner A. C. Mauduit M. Snounou G. (2013). Vaccination using normal live sporozoites under drug treatment. Methods Mol. Biol. 923, 567–576. 10.1007/978-1-62703-026-7_39 22990805

[B154] Rennenberg A. Lehmann C. Heitmann A. Witt T. Hansen G. Nagarajan K. (2010). Exoerythrocytic *Plasmodium* parasites secrete a cysteine protease inhibitor involved in sporozoite invasion and capable of blocking cell death of host hepatocytes. PLoS Pathog. 6:e1000825. 10.1371/journal.ppat.1000825 20361051 PMC2845656

[B155] Rodrigues M. Nussenzweig R. S. Romero P. Zavala F. (1992). The in vivo cytotoxic activity of CD8^+^ T cell clones correlates with their levels of expression of adhesion molecules. J. Exp. Med. 175, 895–905. 10.1084/jem.175.4.895 1372647 PMC2119175

[B156] Rodrigues M. M. Cordey A. S. Arreaza G. Corradin G. Romero P. Maryanski J. L. (1991). CD8^+^ cytolytic T cell clones derived against the *Plasmodium yoelii* circumsporozoite protein protect against malaria. Int. Immunol. 3, 579–585. 10.1093/intimm/3.6.579 1716146

[B157] Roland C. R. Walp L. Stack R. M. Flye M. W. (1994). Outcome of Kupffer cell antigen presentation to a cloned murine Th1 lymphocyte depends on the inducibility of nitric oxide synthase by IFN-gamma. J. Immunol. 153, 5453–5464. 7527442

[B158] Russell J. H. Ley T. J. (2002). Lymphocyte-mediated cytotoxicity. Annu. Rev. Immunol. 20, 323–370. 10.1146/annurev.immunol.20.100201.131730 11861606

[B159] Sanderson N. S. Puntel M. Kroeger K. M. Bondale N. S. Swerdlow M. Iranmanesh N. (2012). Cytotoxic immunological synapses do not restrict the action of interferon-gamma to antigenic target cells. Proc. Natl. Acad. Sci. U.S.A. 109, 7835–7840. 10.1073/pnas.1116058109 22547816 PMC3356634

[B160] Sangani B. Sukumaran P. K. Mahadik C. Yagnik H. Telang S. Vas F. (1990). Thalassemia in Bombay: the role of medical genetics in developing countries. Bull. World Health Organ. 68, 75–81. 2347034 PMC2393015

[B161] Sato T. Yamamoto H. Sasaki C. Wake K. (1998). Maturation of rat dendritic cells during intrahepatic translocation evaluated using monoclonal antibodies and electron microscopy. Cell Tissue Res. 294, 503–514. 10.1007/s004410051201 9799467

[B162] Scheller L. F. Wirtz R. A. Azad A. F. (1994). Susceptibility of different strains of mice to hepatic infection with *Plasmodium berghei*. Infect. Immun. 62, 4844–4847. 7927764 10.1128/iai.62.11.4844-4847.1994 PMC303196

[B163] Schenkel J. M. Fraser K. A. Vezys V. Masopust D. (2013). Sensing and alarm function of resident memory CD8^+^ T cells. Nat. Immunol. 14, 509–513. 10.1038/ni.2568 23542740 PMC3631432

[B164] Schmidt N. W. Butler N. S. Badovinac V. P. Harty J. T. (2010). Extreme CD8 T cell requirements for anti-malarial liver-stage immunity following immunization with radiation attenuated sporozoites. PLoS Pathog. 6:e1000998. 10.1371/journal.ppat.1000998 20657824 PMC2904779

[B165] Schmidt N. W. Butler N. S. Harty J. T. (2011). *Plasmodium*-host interactions directly influence the threshold of memory CD8 T cells required for protective immunity. J. Immunol. 186, 5873–5884. 10.4049/jimmunol.1100194 21460205 PMC3087867

[B166] Schofield L. Ferreira A. Altszuler R. Nussenzweig V. Nussenzweig R. S. (1987a). Interferon-gamma inhibits the intrahepatocytic development of malaria parasites in vitro. J. Immunol. 139, 2020–2025. 2957445

[B167] Schofield L. Villaquiran J. Ferreira A. Schellekens H. Nussenzweig R. Nussenzweig V. (1987b). Gamma interferon, CD8^+^ T cells and antibodies required for immunity to malaria sporozoites. Nature 330, 664–666. 10.1038/330664a0 3120015

[B168] Sedegah M. Finkelman F. Hoffman S. L. (1994). Interleukin 12 induction of interferon gamma-dependent protection against malaria. Proc. Natl. Acad. Sci. U.S.A. 91, 10700–10702. 10.1073/pnas.91.22.10700 7938013 PMC45089

[B169] Seder R. A. Chang L. J. Enama M. E. Zephir K. L. Sarwar U. N. Gordon I. J. (2013). Protection against malaria by intravenous immunization with a nonreplicating sporozoite vaccine. Science 341, 1359–1365. 10.1126/science.1241800 23929949

[B170] Seguin M. C. Klotz F. W. Schneider I. Weir J. P. Goodbary M. Slayter M. (1994). Induction of nitric oxide synthase protects against malaria in mice exposed to irradiated *Plasmodium berghei* infected mosquitoes: involvement of interferon γ and CD8^+^ T cells. J. Exp. Med. 180, 353–358. 10.1084/jem.180.1.353 7516412 PMC2191552

[B171] Seki E. De Minicis S. Gwak G. Y. Kluwe J. Inokuchi S. Bursill C. A. (2009). CCR1 and CCR5 promote hepatic fibrosis in mice. J. Clin. Invest. 119, 1858–1870. 10.1172/JCI37444 19603542 PMC2701864

[B172] Sheth K. Bankey P. (2001). The liver as an immune organ. Curr. Opin. Crit. Care 7, 99–104. 10.1097/00075198-200104000-00008 11373518

[B173] Shi C. Velazquez P. Hohl T. M. Leiner I. Dustin M. L. Pamer E. G. (2010). Monocyte trafficking to hepatic sites of bacterial infection is chemokine independent and directed by focal intercellular adhesion molecule-1 expression. J. Immunol. 184, 6266–6274. 10.4049/jimmunol.0904160 20435926 PMC2921650

[B174] Shin S. C. J. Vanderberg J. P. Terzakis J. A. (1982). Direct infection of hepatocytes by sporozoites of *Plasmodium berghei*. J. Protozool. 29, 448–454. 10.1111/j.1550-7408.1982.tb05431.x 6752394

[B175] Silva J. S. Machado F. S. Martins G. A. (2003). The role of nitric oxide in the pathogenesis of Chagas disease. Front. Biosci. 8, s314–s325. 10.2741/1012 12877141

[B176] Singh A. P. Buscaglia C. A. Wang Q. Levay A. Nussenzweig D. R. Walker J. R. (2007). *Plasmodium* circumsporozoite protein promotes the development of the liver stages of the parasite. Cell 131, 492–504. 10.1016/j.cell.2007.09.013 17981117

[B177] Smith J. E. Alexander J. (1986). Evasion of macrophage microbicidal mechanisms by mature sporozoites of *Plasmodium yoelii* yoelii. Parasitology 93, 33–38. 10.1017/S0031182000049805 3748614

[B178] Spaccapelo R. Naitza S. Robson K. J. H. Crisanti A. (1997). Thrombospondin-related adhesive protein (TRAP) of *Plasmodium berghei* and parasite motility. Lancet 350, 335. 10.1016/S0140-6736(97)24031-6 9251640

[B179] Steers N. Schwenk R. Bacon D. J. Berenzon D. Williams J. Krzych U. (2005). The immune status of Kupffer cells profoundly influences their responses to infectious *Plasmodium berghei* sporozoites. Eur. J. Immunol. 35, 2335–2346. 10.1002/eji.200425680 15997465

[B180] Steinhoff G. (1990). Major histocompatibility complex antigens in human liver transplants. J. Hepatol. 11, 9–15. 10.1016/0168-8278(90)90264-R 2204660

[B181] Steinhoff G. Wonigeit K. Pichlmayr R. (1988). Analysis of sequential changes in major histocompatibility complex expression in human liver grafts after transplantation. Transplantation 45, 394–401. 10.1097/00007890-198802000-00030 2830687

[B182] Steiniger B. Falk P. Van Der Meide P. H. (1988). Interferon-γ in vivo. Induction and loss of class II MHC antigens and immature myelomonocytic cells in rat organs. Eur. J. Immunol. 18, 661–669. 10.1002/eji.1830180502 3132395

[B183] Stewart M. J. Vanderberg J. P. (1988). Malaria sporozoites leave behind trails of circumsporozoite protein during gliding motility. J. Protozool. 35, 389–393. 10.1111/j.1550-7408.1988.tb04115.x 3054075

[B184] Stewart M. J. Vanderberg J. P. (1991). Malaria sporozoites release circumsporozoite protein from their apical end and translocate it along their surface. J. Protozool. 38, 411–421. 10.1111/j.1550-7408.1991.tb01379.x 1787427

[B185] Stewart M. J. Vanderberg J. P. (1992). Electron microscopic analysis of circumsporozoite protein trail formation by gliding malaria sporozoites. J. Protozool. 39, 663–671. 10.1111/j.1550-7408.1992.tb04446.x 1453354

[B186] Stirpe F. Barbieri L. Battelli M. G. Soria M. Lappi D. A. (1992). Ribosome-inactivating proteins from plants: present status and future prospects. Biotechnology 10, 405–412. 10.1038/nbt0492-405 1368484

[B187] Sturm A. Amino R. van de Sand C. Regen T. Retzlaff S. Rennenberg A. (2006). Manipulation of host hepatocytes by the malaria parasite for delivery into liver sinusoids. Science (New York, N.Y.) 313, 1287–1290. 10.1126/science.1129720 16888102

[B188] Sumpter T. L. Abe M. Tokita D. Thomson A. W. (2007). Dendritic cells, the liver, and transplantation. Hepatology 46, 2021–2031. 10.1002/hep.21974 18027875

[B189] Takacs A. C. Swierzy I. J. Luder C. G. (2012). Interferon-gamma restricts *Toxoplasma gondii* development in murine skeletal muscle cells via nitric oxide production and immunity-related GTPases. PLoS ONE 7:e45440. 10.1371/journal.pone.0045440 23024821 PMC3443239

[B190] Tavares J. Formaglio P. Thiberge S. Mordelet E. Van Rooijen N. Medvinsky A. (2013). Role of host cell traversal by the malaria sporozoite during liver infection. J. Exp. Med. 210, 905–915. 10.1084/jem.20121130 23610126 PMC3646492

[B191] Teirlinck A. C. McCall M. B. Roestenberg M. Scholzen A. Woestenenk R. De Mast Q. (2011). Longevity and composition of cellular immune responses following experimental *Plasmodium falciparum* malaria infection in humans. PLoS Pathog. 7:e1002389. 10.1371/journal.ppat.1002389 22144890 PMC3228790

[B192] Todryk S. M. Bejon P. Mwangi T. Plebanski M. Urban B. Marsh K. (2008). Correlation of memory T cell responses against TRAP with protection from clinical malaria, and CD4^+^ CD25^high^ T cells with susceptibility in Kenyans. PLoS ONE 3:e2027. 10.1371/journal.pone.0002027 18446217 PMC2323567

[B193] Trambas C. M. Griffiths G. M. (2003). Delivering the kiss of death. Nat. Immunol. 4, 399–403. 10.1038/ni0503-399 12719728

[B194] Trimnell A. Takagi A. Gupta M. Richie T. L. Kappe S. H. Wang R. (2009). Genetically attenuated parasite vaccines induce contact-dependent CD8^+^ T cell killing of *Plasmodium yoelii* liver stage-infected hepatocytes. J. Immunol. 183, 5870–5878. 10.4049/jimmunol.0900302 19812194

[B195] Trutmann M. Sasse D. (1994). The lymphatics of the liver. Anat. Embryol. (Berl.) 190, 201–209. 10.1007/BF00234299 7818092

[B196] Urban B. C. Ing R. Stevenson M. M. (2005). Early interactions between blood-stage *Plasmodium* parasites and the immune system. Curr. Top. Microbiol. Immunol. 297, 25–70. 10.1007/3-540-29967-X_2 16265902

[B197] Usynin I. Klotz C. Frevert U. (2007). Malaria circumsporozoite protein inhibits the respiratory burst in Kupffer cells. Cell. Microbiol. 9, 2610–2628. 10.1111/j.1462-5822.2007.00982.x 17573905

[B198] Van Braeckel-Budimir N. Harty J. T. (2014). CD8 T-cell-mediated protection against liver-stage malaria: lessons from a mouse model. Front. Microbiol. 5:272. 10.3389/fmicb.2014.00272 24936199 PMC4047659

[B199] Vanderberg J. Mueller A.-K. Heiss K. Goetz K. Matuschewski K. Deckert M. (2007). Assessment of antibody protection against malaria sporozoites must be done by mosquito injection of sporozoites. Am. J. Pathol. 171, 1405–1406; author reply 1406. 10.2353/ajpath.2007.070661 17823294 PMC1988888

[B200] Vanderberg J. P. (2014). Imaging mosquito transmission of *Plasmodium* sporozoites into the mammalian host: immunological implications. Parasitol. Int. 63, 150–164. 10.1016/j.parint.2013.09.010 24060541

[B201] Vanderberg J. P. Chew S. Stewart M. J. (1990). *Plasmodium* sporozoite interactions with macrophages in vitro: a videomicroscopic analysis. J. Protozool. 37, 528–536. 10.1111/j.1550-7408.1990.tb01260.x 2086782

[B202] Vanderberg J. P. Frevert U. (2004). Intravital microscopy demonstrating antibody-mediated immobilization of *Plasmodium berghei* sporozoites injected into skin by mosquitoes. Int. J. Parasitol. 34, 991–996. 10.1016/j.ijpara.2004.05.005 15313126

[B203] van de Sand C. Horstmann S. Schmidt A. Sturm A. Bolte S. Krueger A. (2005). The liver stage of *Plasmodium berghei* inhibits host cell apoptosis. Mol. Microbiol. 58, 731–742. 10.1111/j.1365-2958.2005.04888.x 16238623

[B204] Van Rooijen N. Sanders A. (1996). Kupffer cell depletion by liposome-delivered drugs: comparative activity of intracellular clodronate, propamidine, and ethylenediaminetetraacetic acid. Hepatology 23, 1239–1243. 10.1002/hep.510230544 8621159

[B205] Vaughan A. M. O’Neill M. T. Tarun A. S. Camargo N. Phuong T. M. Aly A. S. (2009). Type II fatty acid synthesis is essential only for malaria parasite late liver stage development. Cell. Microbiol. 11, 506–520. 10.1111/j.1462-5822.2008.01270.x 19068099 PMC2688669

[B206] Vreden S. G. S. Broek M. F. Oettinger M. C. Verhave J. P. Meuwissen J. H. E. T. Sauerwein R. W. (1992). Cytokines inhibit the development of liver schizonts of the malaria parasite *Plasmodium berghei* in vivo. Eur. J. Immunol. 22, 2271–2275. 10.1002/eji.1830220914 1516619

[B207] Wang R. Arevalo-Herrera M. Gardner M. J. Bonelo A. Carlton J. M. Gomez A. (2005). Immune responses to *Plasmodium vivax* pre-erythrocytic stage antigens in naturally exposed Duffy-negative humans: a potential model for identification of liver-stage antigens. Eur. J. Immunol. 35, 1859–1868. 10.1002/eji.200425807 15864779

[B208] Warren A. Le Couteur D. G. Fraser R. Bowen D. G. Mccaughan G. W. Bertolino P. (2006). T lymphocytes interact with hepatocytes through fenestrations in murine liver sinusoidal endothelial cells. Hepatology 44, 1182–1190. 10.1002/hep.21378 17058232

[B209] Weiss W. R. Berzofsky J. A. Houghten R. A. Sedegah M. Hollindale M. Hoffman S. L. (1992). A T cell clone directed at the circumsporozoite protein which protects mice against both *Plasmodium yoelii* and *Plasmodium berghei*. J. Immunol. 149, 2103–2109. 1517574

[B210] WHO. (2013). “World malaria report: 2013,” in WHO Global Malaria Programme. (Geneva: World Health Organization).

[B211] Wiedemann A. Depoil D. Faroudi M. Valitutti S. (2006). Cytotoxic T lymphocytes kill multiple targets simultaneously via spatiotemporal uncoupling of lytic and stimulatory synapses. Proc. Natl. Acad. Sci. U.S.A. 103, 10985–10990. 10.1073/pnas.0600651103 16832064 PMC1544161

[B212] Wilson K. L. Xiang S. D. Plebanski M. (2015). Montanide, Poly I:C and nanoparticle based vaccines promote differential suppressor and effector cell expansion: a study of induction of CD8 T cells to a minimal *Plasmodium berghei* epitope. Front. Microbiol. 6:29. 10.3389/fmicb.2015.00029 25705207 PMC4319470

[B213] Wisse E. De Zanger R. B. Jacobs R. Mccuskey R. S. (1983). Scanning electron microscope observations on the structure of portal veins, sinusoids and central veins in rat liver. Scan. Electron Microsc. 3, 1441–1452. 6648350

[B214] Wuensch S. A. Pierce R. H. Crispe I. N. (2006). Local intrahepatic CD8^+^ T cell activation by a non-self-antigen results in full functional differentiation. J. Immunol. 177, 1689–1697. 10.4049/jimmunol.177.3.1689 16849478

[B215] Yoneyama H. Ichida T. (2005). Recruitment of dendritic cells to pathological niches in inflamed liver. Med. Mol. Morphol. 38, 136–141. 10.1007/s00795-005-0289-0 16170461

[B216] Zarling S. Berenzon D. Dalai S. Liepinsh D. Steers N. Krzych U. (2013). The survival of memory CD8 T cells that is mediated by IL-15 correlates with sustained protection against malaria. J. Immunol. 190, 5128–5141. 10.4049/jimmunol.1203396 23589611 PMC3646969

[B217] Zhang Y. Wang H. Ren J. Tang X. Jing Y. Xing D. (2012). IL-17A synergizes with IFN-gamma to upregulate iNOS and NO production and inhibit chlamydial growth. PLoS ONE 7:e39214. 10.1371/journal.pone.0039214 22745717 PMC3379979

